# Differential microRNA Expression Profiles in Girls with Idiopathic Central Precocious Puberty and Premature Thelarche

**DOI:** 10.3390/ijms27041742

**Published:** 2026-02-11

**Authors:** Hye Jin Lee, Seon Joo Bae, Eu Seon Noh, Hye Young Jin, Il Tae Hwang, Seongho Ryu, Hwal Rim Jeong

**Affiliations:** 1 Department of Pediatrics, Hallym University Kangnam Sacred Heart Hospital, Seoul 07441, Republic of Korea; hjleeped@hallym.or.kr; 2 Soonchunhyang Institute of Medio-Bio Science (SIMS), Department of Integrated Biomedical Sciences, Soonchunhyang University, Cheonan 31151, Republic of Korea; bsj@sch.ac.kr; 3 Department of Pediatrics, Hallym University Kangdong Sacred Heart Hospital, Seoul 05355, Republic of Korea; bbananet1@kdh.or.kr (E.S.N.); hyjin@kdh.or.kr (H.Y.J.); ithwang83@kdh.or.kr (I.T.H.); 4 Department of Pediatrics, Soonchunhyang University College of Medicine, Cheonan 31151, Republic of Korea

**Keywords:** microRNA, precocious puberty, thelarche, puberty

## Abstract

Idiopathic central precocious puberty (CPP) is increasingly observed in girls. Premature thelarche (PT) and exaggerated thelarche (ET) are early pubertal variants that can be challenging to distinguish from CPP in clinical practice. Exosomal microRNAs are stable biomarkers capable of crossing the blood–brain barrier. Although miR-30b-5p has been reported to increase in pubertal boys and girls, human studies investigating microRNAs in CPP and puberty remain limited. To investigate exosomal microRNA expression profiles and associated pathways in early pubertal development, we conducted a cross-sectional study of 28 girls aged 6–8 years. Serum exosomal microRNA expression was analyzed using next-generation sequencing. Differentially expressed microRNAs (DEmiRNAs) between groups were identified, followed by pathway enrichment analysis. Distinct exosomal miRNA expression patterns were observed among the CPP, ET, and control groups, with 307 DEmiRNAs identified. The CPP, PT, and ET groups exhibited distinct miRNA expression profiles compared with the control group. miR-30b-5p was upregulated in the CPP, ET, and PT groups compared with the control group. Pathway enrichment analysis revealed the involvement of various signaling pathways including AGE–RAGE, MAPK, and mTOR signaling pathways. Serum exosomal microRNAs may serve as biomarkers for early puberty and provide insight into metabolic influences on pubertal development.

## 1. Introduction

Central precocious puberty (CPP) is defined as the premature development of secondary sexual characteristics before the age of 8 in girls and 9 in boys, resulting from early activation of the hypothalamic–pituitary–gonadal (HPG) axis. The prevalence and incidence of CPP are approximately 10 times higher in girls than in boys [1,2]. CPP is associated with several adverse outcomes, including reduced final adult height, early menarche in girls, and an increased risk of psychosocial problems, breast cancer, and metabolic disorders such as obesity, hypertension, and type 2 diabetes [3,4,5,6].

CPP is suspected in girls who present with breast budding before the age of 8, along with accelerated growth velocity and advanced bone age (BA) relative to chronological age (CA). The diagnosis is confirmed by a pubertal response in the gonadotropin-releasing hormone (GnRH) stimulation test [7]. Premature thelarche (PT), characterized by breast budding before the age of 8 years without other signs of puberty [8], is often a self-limiting condition requiring no treatment. However, in some cases, particularly between the ages of 6 and 8 years, PT may serve as an early indicator of CPP [9]. Girls with early thelarche and advanced BA but prepubertal gonadotropin response may represent an intermediate stage between PT and CPP within the spectrum of pubertal onset. Although not widely used in clinical practice, the term exaggerated thelarche (ET) is used in this study for clarity [10]. Rare cases of familial CPP have been linked to single-gene mutations such as makorin ring finger protein 3 (*MKRN3)*, the KISS1 metastasis suppressor gene and its receptor, KISS1 receptor (*KISS1* and *KISS1R*), and delta-like noncanonical Notch ligand 1 (*DLK1*) [11], although most cases in girls are idiopathic with no identifiable single cause [12]. Pubertal timing is influenced by factors such as low birth weight, obesity, and endocrine-disrupting chemicals [13], with epigenetics potentially playing a role.

One of the key regulators of epigenetic control, microRNA (miRNA), is a small, noncoding, single-stranded RNA, typically 18–25 nucleotides in length, which suppresses target gene translation at the post-transcriptional level [14]. Several studies have investigated the role of miRNA in the epigenetic regulation of puberty, most of which have been conducted in vitro or in vivo using animal models [15,16,17,18]. However, human data on miRNA and puberty remain limited. Circulating miR-30b-5p levels have been reported to increase during puberty in boys [19,20] and girls [21], and miR-30b-5p is one of the few microRNAs in human studies with a proposed mechanistic link to pubertal regulation through targeting MKRN3, a key inhibitor of GnRH secretion. Additionally, a recent study demonstrated distinct serum miRNA expression patterns among girls with CPP, girls with PT, and controls [22]. 

Tissue-specific expression and limited accessibility of target tissues pose challenges in studying RNA regulation during human pubertal development. Although miRNAs are relatively stable, direct assessment of miRNA changes in central tissues such as the hypothalamus remains difficult. Exosomal miRNA has emerged as a promising and practical solution to overcome these challenges [23]. Exosomes are nanoscale vesicles, typically 30 to 150 nm in size, that mediate intercellular communication by transporting biomolecules such as proteins, lipids, DNA, and RNA between cells. Surrounded by a lipid bilayer, exosomes possess the ability to cross the blood–brain barrier. Since puberty is initiated in the hypothalamus, serum exosomal miRNAs may serve as noninvasive and reliable biomarkers for studying puberty in humans.

Therefore, this study was performed to investigate exosomal miRNA expression profiles in girls with CPP, ET, and PT. Among the differentially expressed miRNAs (DEmiRNAs) identified in this study, miR-30b-5p was further explored as a representative candidate, based on prior human evidence linking miR-30b-5p to pubertal regulation through targeting MKRN3, a key inhibitor of GnRH secretion. Furthermore, we identified the target genes and signaling pathways associated with DEmiRNAs in each group.

## 2. Results

### 2.1. Clinical Characteristics

The clinical characteristics of the total study population (N = 28) are shown in Table 1. The participants’ median age was 7 years, with no significant differences between groups. The Tanner breast stage was 2 in all girls except for those in the CNT group. The median BAs were 6.9, 7.3, 8.5, 9.9, and 10.5 years in the CNT (n = 10), PT (n = 4), ET (n = 4), and CPP (n = 9) groups, respectively. BA was advanced by 2.4 years relative to chronological age in the CPP group and by 1.3 years in the ET group. The height SDS was significantly higher in the CPP group than in the CNT group (1.2 vs. –0.6; adjusted *p* = 0.008). The weight and BMI SDS did not differ between groups. The peak LH level was significantly higher in the CPP group than in the ET group (*p* = 0.002). No significant differences in the peak FSH and E2 levels were observed between the ET and CPP groups. 

### 2.2. Clustering Analysis of Exosomal miRNA Profiles

**Figure 1:**
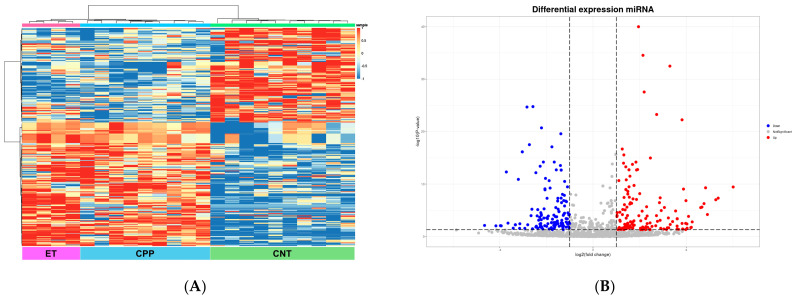
Exosomal miRNA profiles in the CNT, ET, and CPP groups. (**A**) Heatmap showing Z-scores of exosomal miRNAs from the CNT (n = 10), ET (n = 4), and CPP (n = 9) groups, with 174 upregulated (red) and 134 downregulated (blue) miRNAs. (**B**) Volcano plot displaying upregulated (red) and downregulated (blue) DEmiRNAs in the CPP and ET groups compared with the CNT group. CNT, control; ET, exaggerated thelarche; CPP, central precocious puberty.

Serum-derived exosomes were isolated, and exosomal miRNAs were identified using next-generation sequencing, as described in the Section 4. Based on these exosomal miRNA expression profiles, downstream analyses were performed. To identify miRNA expression patterns, a clustering analysis was performed across all groups. Clear separation of clusters was observed among the CPP, ET, and CNT groups, as shown in the heatmap in Figure 1A. In total, 3015 exosomal miRNAs were identified across all groups. After filtering by the thresholds of *p* < 0.05 and |log2 (fold change) | > 1307 miRNAs were differentially expressed between the CPP, ET, and CNT groups. Among these DEmiRNAs, 174 were upregulated and 134 were downregulated in the CPP and ET groups compared with the CNT group (Figure 1B). Table 2 lists the top 10 DEmiRNAs based on the fold change in the CPP and ET groups compared with the CNT group.

**Figure 2:**
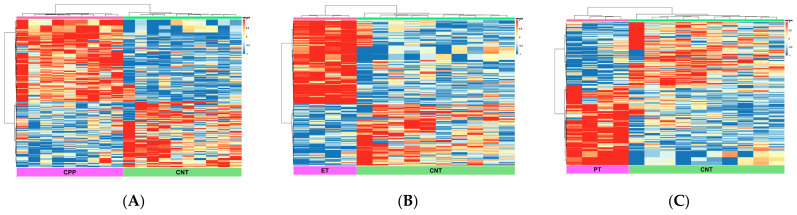
Differentially expressed miRNA profiles of each group compared with controls. Heatmap of differentially expressed exosomal miRNAs in the (**A**) CPP, (**B**) ET, and (**C**) PT groups compared with the CNT group. CPP, central precocious puberty; ET, exaggerated thelarche; PT, premature thelarche; CNT, control.

The clustering of cases in the PT group was not clearly distinct from that in the ET and CPP groups (Supplementary Figure S1). However, each group with pubertal characteristics (CPP, ET, and PT) was clearly separated from the CNT group (Figure 2).

### 2.3. DEmiRNA Profiles of CPP, ET, and PT Groups Compared with CNT Group

To assess DEmiRNA profiles, we conducted a comparative analysis of each group (CPP, ET, ET-ob, and PT) against the CNT group. Significant differences in miRNA profiles were observed between the CPP and CNT groups (Figure 2A). Among the 754 detected miRNAs, 318 were differentially expressed, including 176 upregulated and 142 downregulated miRNAs in the CPP group compared with the CNT group. Table 3 lists the top 10 DEmiRNAs with the highest fold change between the CPP and CNT groups.

The ET group exhibited significant DEmiRNA profiles compared with the CNT group (Figure 2B). In total, 304 miRNAs were differentially expressed (175 upregulated and 129 downregulated miRNAs). Table 4 presents the top 10 DEmiRNAs in the ET group, compared with the CNT group.

The miRNA expression patterns in the PT group were also significantly different from those in the CNT group (Figure 2C). Among 668 detected miRNAs, 279 were differentially expressed (156 upregulated and 123 downregulated). The top 10 DEmiRNAs between the PT and CNT groups are listed in Table 5.

### 2.4. DEmiRNA Profiles Between ET and CPP Groups

**Figure 3:**
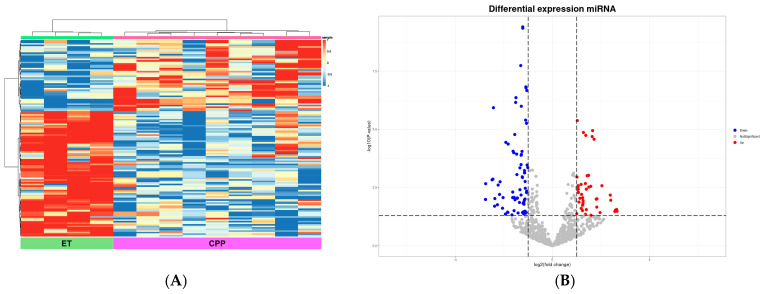
Differentially expressed miRNA profiles of CPP and ET. (**A**) Heatmap of differentially expressed miRNAs in the CPP and ET groups. (**B**) Volcano plot displaying upregulated (red) and downregulated (blue) miRNAs in the CPP group compared with the ET group. CPP, central precocious puberty; ET, exaggerated thelarche.

To identify key miRNAs that differentiate CPP from ET, we directly compared the miRNA expression profiles of the two groups. The heatmap and hierarchical clustering revealed distinct expression differences between CPP and ET (Figure 3). Among the 897 detected miRNAs, 127 were differentially expressed, including 46 upregulated and 81 downregulated in CPP compared with ET. Table 6 lists the top 10 DEmiRNAs based on fold change, including miR-29b-1-5p and miR-137-3p. The complete list of DEmiRNAs is provided in Supplementary Tables (CPP vs. CNT, Table S1; ET vs. CNT, Table S2; PT vs. CNT, Table S3).

### 2.5. Shared and Unique DEmiRNAs Across Groups

**Figure 4:**
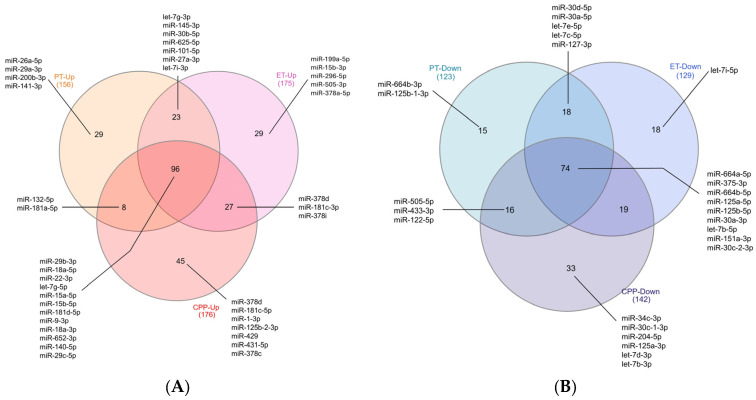
Venn diagram of differentially expressed miRNAs. Venn diagram illustrates the number of differentially expressed and shared (**A**) upregulated and (**B**) downregulated miRNAs in the CPP, ET, and PT groups compared with the control group. miRNAs previously associated with puberty are indicated. CPP, central precocious puberty; ET, exaggerated thelarche; PT, premature thelarche.

A Venn diagram was constructed using the lists of DEmiRNAs from the CPP, ET, and PT groups compared with the CNT group (Figure 4). In total, 96 miRNAs were commonly upregulated across all three groups, including miR-29b-3p, miR-18a-5p, miR-22-3p, let-7g-5p, miR-15a-5p, miR-15b-5p, miR-181d-5p, miR-9-3p, miR-18a-3p, miR-652-3p, miR-140-5p, and miR-29c-5p. Additionally, 45 miRNAs were exclusively upregulated in the CPP group, including miR-378d, miR-181c-5p, miR-1-3p, miR-125b-2-3p, miR-429, miR-431-5p, and miR-378c, while 29 miRNAs were uniquely upregulated in the ET and PT groups (Figure 4A).

With respect to downregulated miRNAs, 74 were commonly downregulated across all three groups, including miR-664a-5p, miR-375-3p, miR-664b-5p, miR-125a-5p, miR-125b-5p, miR-30a-3p, let-7b-5p, miR-151a-3p, and miR-30c-2-3p. Additionally, 33 miRNAs were uniquely downregulated in the CPP group, including miR-34c-3p, miR-30c-1-3p, miR-204-5p, miR-125a-3p, let-7d-3p, and let-7b-3p, while 18 were uniquely downregulated in the ET group and 15 in the PT group (Figure 4B).

### 2.6. Upregulation of miR-30b-5p Across All Groups

**Figure 5:**
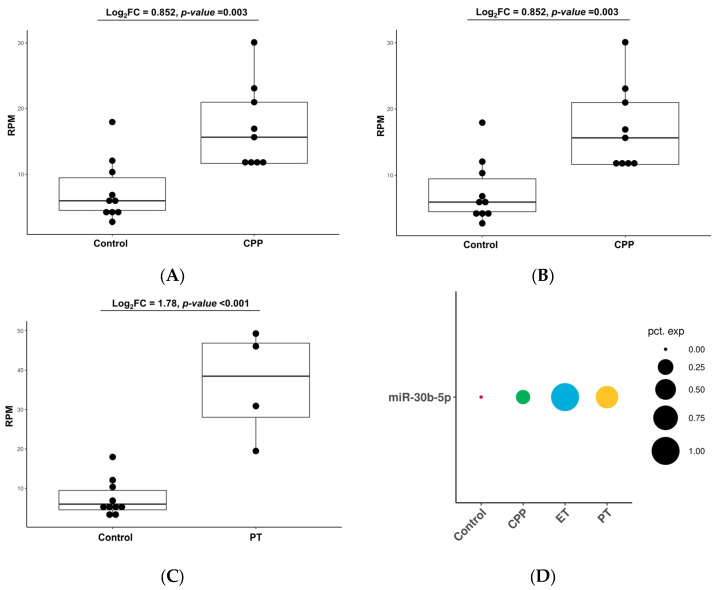
Upregulation of miR-30b-5p across all groups. Box plots show higher RPM levels of miR-30b-5p in the (**A**) CPP, (**B**) ET, and (**C**) PT groups compared with the control group. (**D**) Schematic diagram illustrates the average RPM percentage expression of miR-30b-5p in each comparative analysis. RPM, read per million; FC, fold change; CPP, central precocious puberty; ET, exaggerated thelarche; PT, premature thelarche.

Comparative analyses revealed consistent upregulation of miR-30b-5p across the CPP, ET, and PT groups compared with the CNT group (Figure 5). The log2 (fold change) values for ET and PT were 2.4 and 1.8, respectively, with *p*-values < 0.001. The CPP group showed a log2 (fold change) of 0.85, indicating approximately 1.8-fold higher expression of miR-30b-5p compared with CNT (*p* = 0.003). The average reads per million percentage expression of miR-30b-5p in each group is shown in Figure 5D.

### 2.7. Target Genes and Pathway Enrichment Analysis of DEmiRNAs

**Figure 6:**
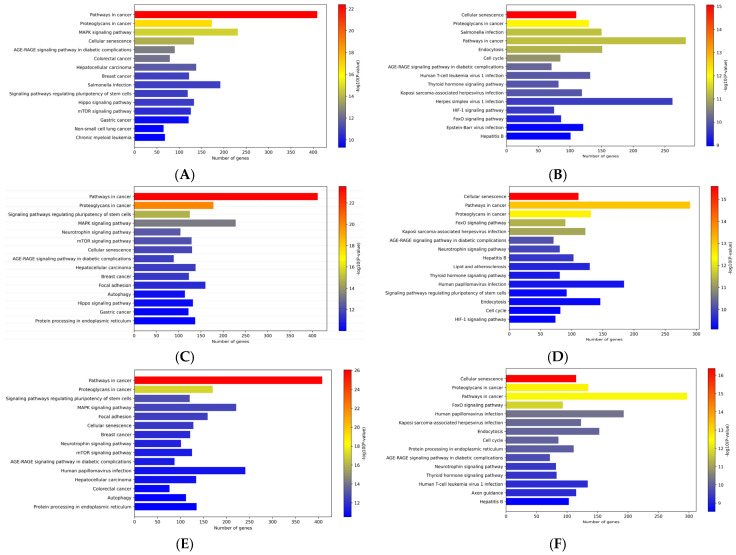
Enriched pathways of predicted target genes of differentially expressed miRNAs. Top 15 enriched pathways identified using Enrichr, ranked by significance. Pathways enriched in (**A**) upregulated and (**B**) downregulated miRNAs in CPP, (**C**) upregulated and (**D**) downregulated miRNAs in ET, and (**E**) upregulated and (**F**) downregulated miRNAs in PT are shown. CPP, central precocious puberty; ET, exaggerated thelarche; PT, premature thelarche.

Pathway enrichment analyses were conducted using the predicted target genes of all differentially expressed microRNAs (DEmiRNAs) identified in this study (Figure 6). In the CPP group compared with the CNT group, a total of 11,356 predicted target genes were identified as potentially regulated by upregulated miRNAs, whereas 7766 predicted target genes were identified as potentially regulated by downregulated miRNAs. The advanced glycation end product (AGE)–receptor for AGE (RAGE), mitogen-activated protein kinase (MAPK), Hippo, mammalian target of rapamycin (mTOR), hypoxia-inducible factor 1 (HIF-1), and forkhead box O (FoxO) signaling pathways were among the canonical pathways significantly associated with the target genes of DEmiRNAs in the CPP and CNT groups (Figure 6A,B). In the ET group compared with the CNT group, 11,367 target genes were predicted for upregulated miRNAs, and 7780 for downregulated miRNAs, which were associated with pathways including MAPK, neurotrophin, mTOR, AGE–RAGE, Hippo, FoxO, and HIF-1 signaling (Figure 6C,D). Similarly, 10,955 and 8130 target genes were identified for upregulated and downregulated miRNAs, respectively, in the PT group compared with the CNT group, which were associated with the MAPK, neurotrophin, mTOR, AGE–RAGE, and FoxO signaling pathways (Figure 6E,F). Notably, the MAPK, mTOR, FoxO, neurotrophin, and AGE–RAGE signaling pathways were consistently enriched across the CPP, ET, and PT groups compared with the CNT group.

## 3. Discussion

In this study, we identified distinct exosomal miRNA profiles in girls with CPP, ET, and PT compared with the CNT group. Hierarchical clustering analysis revealed clear separation between the CNT group and the other groups, suggesting that exosomal miRNAs may serve as potential biomarkers for pubertal development. Notably, previously reported miRNAs associated with puberty, including miR-30b-5p, exhibited differential expression between groups, alongside numerous DEmiRNAs not yet linked to puberty or CPP. Pathway enrichment analysis highlighted the involvement of various biological pathways, with the AGE–RAGE signaling pathway showing strong relevance across all groups. These findings suggest a potential role for exosomal miRNAs in early pubertal development.

Puberty is initiated by activation of the HPG axis. This involves pulsatile secretion of GnRH from the hypothalamus, which stimulates the pituitary gland to release LH and FSH. These hormones promote gonadal maturation, sex hormone production, and the development of secondary sexual characteristics. CPP is associated with full activation of the HPG axis, whereas ET may involve minimal or inconsistent activation, often insufficient to meet the diagnostic threshold for CPP. In our study, clear differences in miRNA expression profiles were observed between the CPP and ET groups. These differences are interpreted in the context of their clinical classification, which is defined by the presence or absence of a pubertal response on the GnRH stimulation test, rather than as evidence of direct mechanistic regulation of HPG axis activity. By contrast, the PT group, which was characterized by isolated breast budding without BA acceleration, exhibited a distinct miRNA expression pattern compared with the CNT group but was not clearly separated from the CPP and ET groups. The follow-up of the PT and ET groups in this study revealed that all participants progressed toward early puberty. These findings raise the possibility that PT, ET, and CPP might lie along a spectrum of pubertal progression, with differences potentially reflecting the degree and timing of HPG axis activation, rather than entirely separate biological mechanisms.

We identified numerous DEmiRNAs across groups that target genes involved in pubertal timing or are associated with CPP in animal models and in vitro experiments. LIN28B, a gene known for its role in pubertal timing, has been shown to exhibit reduced expression associated with HPG axis activation and the onset of puberty [24]. LIN28B and the let-7 miRNA family inhibit each other: LIN28B blocks let-7 differentiation, while let-7 members suppress LIN28B expression [25]. In our study, let-7g-5p was upregulated in the CPP, ET, and PT groups, while let-7g-3p and let-7i-3p were upregulated in the ET and PT groups, aligning with previous studies. By contrast, let-7b, let-7c-5p, let-7e-5p, and let-7i-5p were downregulated in the CPP, ET, or PT groups, which warrants further investigation.

miR-132 and miR-9 have been reported to inhibit LIN28B [26]. In our study, miR-9 was upregulated in all three groups (CPP, ET, and PT), and miR-132 was upregulated in the CPP and PT groups, consistent with previous findings [26]. miR-145 is known to suppress the expression of c-Myc, a gene that activates LIN28B in cancer cells [27,28]. Given the role of LIN28B in pubertal timing, the upregulation of miR-145 may contribute to early puberty by inhibiting this pathway. Our results showed that miR-145 was upregulated in the PT and ET groups, supporting this hypothesis. 

The miR-200 family, including miR-429, is highly expressed in GnRH neurons in mice and has been reported to increase in GnRH cells around the onset of puberty [18]. In line with these findings, our study observed an upregulation of miR-429 in the CPP group and miR-200b in the PT group. While the upregulation of miR-200/429 in CPP is consistent with prior evidence that this family enhances LH secretion via Zeb1 inhibition [18], its clinical relevance in PT is unclear, given that PT generally does not involve increased LH levels.

We also observed that miR-664 was downregulated, aligning with previous studies showing that lower levels of miR-664-2 were associated with precocious puberty [29,30,31]. Suppression of miR-664 in rat hypothalamic neurons has been shown to increase NMDAR1 protein expression, advancing puberty onset [29]. Additionally, miR-15a is known to inhibit DLK1 [30], and DLK1 deficiency has been associated with the development of CPP [31]. Thus, increased miR-15a levels may suppress DLK1, potentially leading to earlier puberty onset. Consistent with this hypothesis, our study showed that miR-15a was upregulated in the CPP, ET, and PT groups supporting its possible involvement in early pubertal activation.

Animal studies have shown that miR-29, which is present in the pituitary, plays a critical role in brain maturation [32], increases in serum levels as puberty approaches [16], and is elevated in the ovary during the follicular phase [33]. In our study, miR-29a was upregulated across the CPP, ET, and PT groups, aligning with previous findings. However, one study showed that deletion of miR-29 led to an increase in Gnrh1 expression and earlier puberty onset in mice, presenting results that contradict our findings, where CPP, ET, and PT groups exhibited upregulated miR-29a compared with the CNT group [15]. Notably, we also observed that miR-29b-1-5p was downregulated in the CPP group compared with the ET group, suggesting a potential association between miR-29b and HPG axis activation. Although the miR-29 family appears to be linked to pubertal onset, further research is required to clarify its precise mechanisms.

Some DEmiRNAs in this study exhibited expression patterns opposite to those reported in previous research. For instance, miR-375 was downregulated across the CPP, ET, and PT groups, whereas prior studies have reported that miR-375 indirectly increases Gnrh gene expression [34]. Similarly, while miR-505-3p has been shown to suppress puberty in female mice [35], our study showed that miR-505-3p was upregulated in the ET group and that miR-505-5p was upregulated in the CPP and PT groups. Additionally, miR-199-3p and miR-137-3p have been reported to suppress Kiss1 gene expression, thereby delaying puberty onset [36,37]. However, in our study, miR-199-3p was upregulated in the ET group, and miR-137-3p was upregulated in the CPP group compared with the ET group, findings that conflict with prior reports. These discrepancies may be due to differences in the tissues examined or variations in miRNA function depending on species, sex, or the involvement of complex regulatory pathways. Further research is needed to elucidate these mechanisms.

While some DEmiRNAs identified in this study have previously been associated with puberty, the majority have not yet been linked to pubertal mechanisms. To explore their potential roles, target genes were identified, and pathway enrichment analysis was conducted. The MAPK, mTOR, and AGE–RAGE signaling pathways were enriched across the CPP, PT, and ET groups compared with the CNT group. Both the MAPK and mTOR signaling pathways are known to stimulate GnRH secretion by increasing Kiss1 expression, which is associated with pubertal onset [36,38,39]. 

Although no direct evidence links the AGE–RAGE pathway to pubertal onset, it is known to regulate the MAPK pathway [40,41], suggesting a possible role in pubertal activation. AGEs are reactive sugar-derived metabolites formed through metabolism, oxidative stress, or dietary intake [42]. AGEs are abundant in processed foods, as well as in food fried or grilled with sugar [43]. Given that adequate energy availability is essential for puberty, metabolic signals such as AGE–RAGE may influence HPG axis activation. The AGE–RAGE pathway has also been implicated in breast tissue development and breast cancer, with studies showing that high AGE intake promotes mammary gland growth in mice [44]. Thus, the enrichment of the AGE–RAGE signaling pathway in groups with breast budding may contribute to both central HPG axis activation and direct effects on breast development. The increasing consumption of processed and sugar-rich foods among children may contribute to the rise in idiopathic CPP and early puberty, potentially involving this pathway. Further research is needed to elucidate the precise mechanisms by which metabolic factors, exosomal miRNAs, and HPG axis activation interact in pubertal progression.

The Hippo signaling pathway was enriched in the CPP. While this pathway is primarily known for regulating cell proliferation and differentiation [45], its role in pubertal onset has not yet been established. However, differences in the expression of Hippo signaling pathway genes have been observed in male rats before and after puberty, suggesting its involvement in Sertoli cell function [46]. The neurotrophin signaling pathway was enriched in the ET and PT groups. Although its direct association with pubertal onset remains unclear, ovarian neurotrophins are known to promote follicular development and ovulation, with excessive activity linked to polycystic ovarian syndrome [47]. In the ET group, the FoxO and HIF-1 signaling pathways were enriched, both of which are associated with energy metabolism. While no direct evidence links these pathways to pubertal onset, FoxO plays a role in ovarian follicular development [48], and under hypoxic conditions, FoxO interacts with the HIF-1 pathway [49].

Collectively, the predicted target genes of the DEmiRNAs converge on molecular pathways that are biologically coherent with early activation of the hypothalamic–pituitary–gonadal axis. Many of these targets participate in neuronal development and synaptic plasticity (e.g., axon guidance, MAPK, PI3K–Akt signaling), which are essential for the maturation and excitability of GnRH neurons. Others are linked to metabolic and growth-related pathways (e.g., insulin, mTOR, AMPK), aligning with the well-recognized permissive role of metabolic cues in pubertal initiation. Several genes also regulate steroidogenesis, gonadotropin signaling, and hormone receptor activity, suggesting potential peripheral contributions to pubertal acceleration. 

Taken together, these gene networks outline a mechanistic framework in which dysregulated exosomal miRNAs may influence the neuroendocrine nodes that control GnRH release—either by attenuating inhibitory inputs (e.g., MKRN3-related mechanisms) or by enhancing stimulatory pathways involved in neuronal activation, metabolic sensing, and hormone signaling. Although causality cannot be inferred from these data, the collective functional profile of these genes is consistent with biological processes that, if perturbed, could favor the premature onset of puberty.

We also identified significantly higher expression levels of exosomal miR-30b-5p in the CPP, ET, and PT groups compared with the CNT group. Previous studies have reported increased miR-30b expression in pubertal boys [19,20] and girls [21], suggesting a conserved role across genders. miR-30b is known to regulate the expression of MKRN3, a gene that inhibits the HPG axis, supporting its involvement in early pubertal activation. [20,50]. Therefore, increased miR-30b in girls with CPP, ET, and PT may reduce MKRN3 expression, subsequently activating the HPG axis and leading to early sexual maturation.

To our knowledge, this is the first study to comprehensively characterize exosomal miRNA profiles and their potential regulatory pathways in girls with early signs of puberty, providing novel insights into the molecular mechanisms underlying pubertal progression. However, this study has several limitations, including its cross-sectional design, which prevents the establishment of causal relationships. Although prior studies have shown hypothalamic miRNA involvement in puberty in animal models, the origin of circulating miRNAs remains uncertain. It is possible that the serum miRNA profiles observed in our study reflect not only central neuroendocrine changes but also peripheral responses in the gonads or other tissues activated through the gonadotropic axis. Given that PT and ET can progress to CPP, a longitudinal approach would have been more informative in assessing whether miRNAs predict pubertal progression. and a small sample size, which may introduce intra- and inter-individual variations. A larger, longitudinal cohort study is necessary to validate our findings and determine whether exosomal miRNAs can serve as reliable biomarkers for early puberty. In addition, the lack of independent experimental validation limits the interpretability of the identified miRNA signatures, which should therefore be regarded as hypothesis-generating. Future studies using larger, longitudinal cohorts with targeted validation will be required to confirm their biological and clinical relevance. 

Despite these limitations, this study provides an important first step toward understanding exosomal miRNA dynamics in early pubertal development. Our findings highlight the potential of circulating exosomal miRNAs as noninvasive markers of early HPG-axis activation and establish a conceptual foundation for future mechanistic and biomarker-oriented research. Continued investigation using longitudinal designs, multi-omics integration, and functional studies will be crucial to delineate the precise roles of these miRNAs in the timing and progression of puberty.

## 4. Materials and Methods

### 4.1. Study Design and Participants

This cross-sectional case–control study included girls aged 6–8 years with or without pubertal characteristics. Serum exosomal miRNA expression profiles were analyzed across groups based on clinical pubertal characteristics. Additionally, target gene prediction and pathway enrichment analysis were performed. 

In total, 28 girls under the age of 9 were enrolled in this study. The girls had no underlying medical conditions and had visited the outpatient clinic at Soonchunhyang University Cheonan Hospital or Kangdong Sacred Heart Hospital for growth and puberty evaluations.

**Figure 7:**
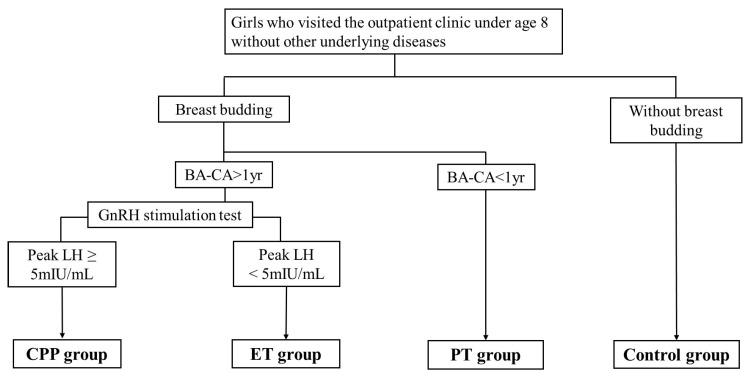
Flow diagram of the study population. CPP, central precocious puberty; ET, exaggerated thelarche; PT, premature thelarche; BA, bone age; CA, chronological age; GnRH, gonadotropin-releasing hormone; LH, luteinizing hormone. Obese and overweight children were excluded.

We categorized the participants into five groups based on their clinical characteristics (Figure 7). The CPP group consisted of girls with breast budding before the age of 8, advanced BA compared to CA, and a pubertal response on the GnRH stimulation test (peak luteinizing hormone [LH] level ≥ 5 mIU/mL). The ET group included girls with breast budding before the age of 8 and advanced BA compared with CA but with a prepubertal response on the GnRH stimulation test (peak LH level < 5 mIU/mL). The PT group consisted of girls with breast budding before the age of 8 without advanced BA. The control (CNT) group consisted of girls without breast budding. The control group consisted of prepubertal girls with normal height and weight for age and no evidence of other endocrine disorders. Children who were overweight or obese (BMI ≥ 85th percentile) were excluded from all groups. Participants with a history of preterm birth or who were born small for gestational age were also excluded.

### 4.2. Anthropometric and Biochemical Measurements

Height was measured using a Harpenden stadiometer (Holtain Ltd., Wales, UK), and weight was measured using a digital scale (150 A; Cas Co., Ltd., Seoul, Republic of Korea). BMI was calculated as weight (kg) divided by height squared (m^2^). Height, weight, and BMI standard deviation scores (SDS) and percentiles were determined for each participant based on the 2017 Korean National Growth Charts [51]. Tanner breast staging and BA assessment using the Greulich and Pyle method were performed by two pediatric endocrinologists (H.R. Jeong and I.T. Hwang).

The GnRH stimulation test was conducted in girls with breast budding before the age of 8 and advanced BA. The levels of LH and follicle-stimulating hormone (FSH) were measured at baseline and at 30, 45, 60, and 90 min after intravenous administration of 0.1 mg LH-releasing hormone (100 μg Relefact; Sanofi-Aventis, Paris, France). The serum levels of LH, FSH, and estradiol (E2) were determined using electrochemiluminescence immunoassays on Cobas e 801 and e 402 analyzers (Roche Diagnostics, Mannheim, Germany), with an analytical sensitivity of 5 pg/mL for E2 and 0.3 IU/L for LH and FSH.

### 4.3. Exosomal miRNA Sequencing

Venous serum samples from each participant were immediately stored at −80 °C until analysis. Exosomes were isolated from 1-mL serum samples using the miRCURY Exosome Serum/Plasma Kit (QIAGEN, Hilden, Germany) according to the manufacturer’s instructions. Briefly, to remove cells and debris, human serum was centrifuged at 3000× *g* for 10 min. Next, 400 µL of Precipitation Buffer A was added, and the mixture was incubated at 4 °C. Phosphate-buffered saline was used to resuspend the pellets obtained after centrifugation at 1500× *g* for 30 min at 20 °C.

An miRNeasy Serum/Plasma Kit (QIAGEN) was used to extract miRNAs from exosomes according to the manufacturer’s instructions, and the resulting miRNAs were eluted in 20 µL of RNase-free water. miRNA characterization and quality assessment were performed using the High Sensitivity RNA ScreenTape assay with the 4150 TapeStation System (Agilent Technologies, Santa Clara, CA, USA).

Next-generation sequencing was performed for exosomal miRNA. A total of 1 ng of extracted miRNA was used as an input for each library. Small RNA libraries were constructed using the QIAseq miRNA Library Kit (QIAGEN) following the manufacturer’s instructions. Adapter ligation, complementary DNA synthesis, and polymerase chain reaction amplification were used to generate the sequencing libraries.

The libraries were purified using a magnetic bead-based method and validated for size, purity, and concentration using the Agilent 4150 TapeStation System (Agilent Technologies). Equimolar amounts of the libraries were pooled and templated on an Ion Chef instrument (Thermo Fisher Scientific, Waltham, MA, USA) to generate Ion 550 Chips loaded with template-positive Ion Sphere Particles. Sequencing was performed using an Ion S5 Sequencer (Thermo Fisher Scientific). Torrent Suite Software (Thermo Fisher Scientific, version 5.16.1) was used to analyze sequencing data. Using miRDeep2, clustered reads were aligned to reference miRNA sequences from miRBase (version 22) to identify and profile miRNAs.

### 4.4. Analysis of Differential miRNA Expression

Analysis of DEmiRNAs was performed to identify miRNAs that were either upregulated or downregulated in exosomes from serum samples of each group. Read counts were normalized using the trimmed mean of M-values method in the edgeR statistical software package (Bioconductor, version 3.38.4; http://bioconductor.org/). For each miRNA, expression values were compared among groups using a generalized linear model approach, with differential expressions assessed through likelihood ratio tests. DEmiRNAs between two groups were identified using edgeR, while those among three groups were analyzed using the DESeq2 package (Bioconductor). Differential expressions were determined based on a |log2 (fold change) | ≥ 1 (equivalent to |fold change| ≥ 2) and *p*-values < 0.05. Hierarchical clustering analysis was performed using complete linkage and Euclidean distance to visualize the expression patterns of DEmiRNAs that met the specified criteria. Normalized expression values were visualized using dot plots generated with the ggplot2 package, adhering to the principles of the grammar of graphics. Venn diagrams were created using the InteractiveVenn tool (https://www.interactivenn.net/) [52].

### 4.5. Target Gene Prediction and Pathway Enrichment Analysis 

To identify potential target genes of the DEmiRNAs, we utilized the TargetScan database integrated within miRTargetLink 2.0. To explore the biological pathways potentially influenced by the DEmiRNAs and their predicted target genes, pathway enrichment analysis was conducted using the Kyoto Encyclopedia of Genes and Genomes (KEGG) Human 2021 database with the enrichR tool (version 3.38.4) [53]. Pathways with significant enrichment were identified based on *p*-values < 0.05.

### 4.6. Statistical Analysis 

Comparisons of clinical characteristics among groups were performed using the Mann–Whitney U test for two-group comparisons and the Kruskal–Wallis test for comparisons among three or more groups, followed by Bonferroni-corrected post hoc analysis. All analyses were conducted using SPSS Statistics software version 28.0 (IBM Corp., Armonk, NY, USA) and R software version 4.1.0 (R Development Core Team, Vienna, Austria; https://cran.r-project.org/). A *p*-value < 0.05 was considered statistically significant.

## 5. Conclusions

This study identified distinct serum exosomal miRNA expression patterns among girls in the CPP, ET, and PT groups compared with the CNT group. Notably, miR-30b-5p was consistently upregulated in all groups with breast budding, reinforcing its potential role in pubertal initiation. The enrichment of the AGE–RAGE signaling pathway across these groups suggests that metabolic and dietary factors may influence pubertal timing, possibly through interactions with the HPG axis. These findings suggest that serum exosomal miRNAs could serve as valuable biomarkers for early puberty and may offer new insights into the influence of metabolism on pubertal development. Further research is warranted to explore their potential as diagnostic or therapeutic targets for CPP.

## Figures and Tables

**Table 1:** Clinical characteristics of the total study population.

	CNT (n = 10)	PT (n = 4)	ET (n = 4)	CPP (n = 9)	*p*-Value
Age (yr)	7.3 (6.9, 7.8)	6.8 (6.1, 7.3)	7.3 (7.0, 7.5)	7.9 (7.7, 8.2)	0.050
Tanner stage					<0.001 ^a^
I	10 (100)	0 (0)	0 (0)	0 (0)	
II	0 (0)	4 (100)	4 (100)	9 (100)	
BA (yr)	6.9 (6.9, 7.4)	7.3 (6.6, 7.8)	8.5 (8.3, 8.9)	10.5 (9.8, 10.8)	0.001 ^b^
∆(BA-CA)	−0.03(−0.4, 0.3)	0.5 (0.5, 0.6)	1.3 (1.1, 1.6)	2.4 (1.7, 2.9)	<0.001 ^c^
Height (cm)	119.4 (115.6, 122.1)	118.1 (117.7, 118.4)	122.3 (120.3, 124.2)	132.3 (128.0, 134.0)	0.001 ^b^
Weight (kg)	23.5 (20.0, 24.4)	23.0 (21.9, 23.8)	23.8 (21.5, 26.6)	27.5 (25.2, 31.8)	0.007 ^c^
BMI (kg/m^2^)	16.0 (15.6, 16.4)	16.4 (15.7, 17.1)	16.2 (15.2, 17.2)	16.8 (14.9, 18.1)	0.736
Height SDS	−0.6 (−0.8, −0.3)	−0.3 (−1.0, 0.7)	−0.3 (−0.5, 0.2)	1.2 (1.0, 1.4)	0.015 ^c^
Weight SDS	−0.3 (−0.7, −0.2)	0.1 (−0.7, 0.7)	−0.2 (−0.7, 0.5)	0.1 (−0.1, 1.3)	0.2
BMI SDS	−0.1 (−0.4, 0.2)	0.6 (−0.3, 1.4)	−0.1 (−0.6, 0.5)	0.2 (−0.9, 0.8)	0.681
Peak LH (IU/L)	NA	NA	3.8 (3.5, 4.2)	7.9 (6.8, 13.4)	0.002
Peak FSH (IU/L)	NA	NA	14.0 (12.3, 15.8)	14.4 (11.7, 17.1)	0.825
Peak E2 (pg/mL)	NA	NA	3.5 (3.5, 4.5)	12.5 (3.5, 27.2)	0.105

Values are presented as median with interquartile range (Q1, Q3) or n (%). *p*-values of Kruskal–Wallis test with Bonferroni post hoc analysis are presented. Significant differences in pairwise post hoc analysis with Mann–Whitney U test with Bonferroni correction between two groups are annotated as follows: ^a.^ *p* < 0.05 for CPP vs. CNT, ET vs. CNT, and PT vs. CNT; ^b.^ *p* < 0.05 for CPP vs. CNT and CPP vs. PT; ^c.^ *p* < 0.05 for CPP vs. CNT. CNT, control group; PT, premature thelarche group; ET, exaggerated thelarche group; CPP, central precocious puberty group; BA, bone age; CA, chronological age; BMI, body mass index; SDS, standard deviation score; LH, luteinizing hormone; FSH, follicle-stimulating hormone; E2, estradiol.

**Table 2:** Top 10 DEmiRNAs in the CPP and ET groups (vs. CNT group) according to fold change.

miRNA	log_2_ (Fold Change)	*p*-Value
hsa-miR-548e	6.01	<0.001
hsa-miR-20a-3p	5.37	<0.001
hsa-miR-378d	5.27	<0.001
hsa-miR-1537-3p	4.91	<0.001
hsa-miR-369-3p	4.83	<0.001
hsa-miR-378d	4.76	<0.001
hsa-miR-1285-3p	4.67	<0.001
hsa-miR-495-3p	4.26	0.002
hsa-miR-34c-5p	4.22	0.017
hsa-miR-4779	4.20	0.021
hsa-miR-6782-3p	−3.15	0.028
hsa-miR-1468-5p	−3.20	<0.001
hsa-miR-6859-3p	−3.33	0.006
hsa-miR-4492	−3.42	0.026
hsa-miR-1236-5p	−3.65	0.003
hsa-miR-4516	−3.72	<0.001
hsa-miR-365a-5p	−3.94	0.009
hsa-miR-6779-5p	−3.97	0.009
hsa-miR-4667-5p	−4.17	0.009
hsa-miR-6869-5p	−4.65	0.008

DEmiRNAs, differentially expressed miRNAs; CPP, central precocious puberty; ET, exaggerated thelarche; CNT, control.

**Table 3:** Top 10 DEmiRNAs in the CPP group (vs. CNT group) according to fold change.

miRNA	log_2_ (Fold Change)	*p*-Value
hsa-miR-369-3p	4.56	<0.001
hsa-miR-548e-3p	4.25	<0.001
hsa-miR-374a-5p	3.95	<0.001
hsa-miR-374b-5p	3.78	<0.001
hsa-miR-17-3p	3.76	<0.001
hsa-miR-378d	3.69	<0.001
hsa-miR-20a-3p	3.67	<0.001
hsa-miR-378d	3.43	<0.001
hsa-miR-4429	3.28	<0.001
hsa-miR-628-5	3.05	<0.001
hsa-miR-6891-5p	−2.52	<0.001
hsa-miR-3605-5p	−2.61	<0.001
hsa-miR-1236-5p	−2.61	0.002
hsa-miR-6859-3p	−2.63	0.002
hsa-miR-1224-5p	−2.74	<0.001
hsa-miR-129-5p	−2.83	<0.001
hsa-miR-365a-5p	−3.02	<0.001
hsa-miR-6779-5p	−3.08	<0.001
hsa-miR-1468-5p	−3.15	<0.001
hsa-miR-4516	−3.61	<0.001

DEmiRNAs, differentially expressed miRNAs; CPP, central precocious puberty; CNT, control.

**Table 4:** Top 10 DEmiRNAs in the ET group (vs. CNT group) according to fold change.

miRNA	log_2_ (Fold Change)	*p*-Value
hsa-miR-374a-5p	4.41	<0.001
hsa-miR-18a-5p	4.10	<0.001
hsa-miR-4429	4.06	<0.001
hsa-miR-374b-5p	3.98	<0.001
hsa-miR-7-1-3p	3.84	<0.001
hsa-miR-20a-5p	3.65	<0.001
hsa-miR-22-5p	3.62	<0.001
hsa-miR-4433a-3p	3.44	<0.001
hsa-miR-106b-5p	3.42	<0.001
hsa-miR-369-3p	3.31	<0.001
hsa-miR-1275	−3.19	0.010
hsa-miR-3154	−3.21	0.001
hsa-miR-206	−3.29	<0.001
hsa-miR-129-5p	−3.41	<0.001
hsa-miR-4511	−3.41	0.003
hsa-miR-1224-5p	−3.50	<0.001
hsa-miR-6807-5p	−3.52	0.002
hsa-miR-7704	−3.63	0.001
hsa-miR-4516	−3.98	<0.001
hsa-miR-1299	−4.32	0.001

DEmiRNAs, differentially expressed miRNAs; ET, exaggerated thelarche; CNT, control.

**Table 5:** Top 10 DEmiRNAs in the PT group (vs. CNT group) according to fold change.

miRNA	log_2_ (Fold Change)	*p*-Value
hsa-miR-22-5p	4.41	<0.001
hsa-miR-31-5p	4.31	<0.001
hsa-miR-374a-5p	3.54	<0.001
hsa-miR-374b-5p	3.53	<0.001
hsa-miR-4429	3.49	<0.001
hsa-miR-499a-5p	3.20	<0.001
hsa-miR-20a-5p	3.15	<0.001
hsa-miR-222-3p	3.14	<0.001
hsa-miR-7-1-3p	3.09	<0.001
hsa-miR-106b-5p	3.02	<0.001
hsa-miR-4750-5p	−3.32	<0.001
hsa-miR-206	−3.34	<0.001
hsa-miR-129-5p	−3.43	<0.001
hsa-miR-6514-5p	−3.58	0.003
hsa-miR-6891-5p	−3.58	0.001
hsa-miR-4669	−3.65	<0.001
hsa-miR-7704	−3.71	<0.001
hsa-miR-30c-2-3p	−4.36	<0.001
hsa-miR-1343-3p	−4.93	<0.001
hsa-miR-885-3p	−5.12	<0.001

DEmiRNAs, differentially expressed miRNAs; PT, premature thelarche; CNT, control.

**Table 6:** Top 10 DEmiRNAs in the CPP group (vs. ET group) according to fold change.

miRNA	log2 (Fold Change)	*p*-Value
hsa-miR-6729-5p	2.66	0.033
hsa-miR-1275	2.64	0.028
hsa-miR-137-3p	2.60	0.030
hsa-miR-6772-3p	2.57	0.034
hsa-miR-10399-3p	2.40	0.011
hsa-miR-1299	2.38	0.007
hsa-miR-1246	2.04	0.003
hsa-miR-4766-3p	1.96	0.037
hsa-miR-3065-5p	1.83	0.010
hsa-miR-6805-5p	1.83	0.022
hsa-miR-204-3p	−2.18	0.006
hsa-miR-885-5p	−2.24	0.003
hsa-miR-29b-1-5p	−2.26	0.017
hsa-miR-511-5p	−2.37	0.009
hsa-miR-3117-3p	−2.38	0.020
hsa-miR-5010-5p	−2.43	<0.001
hsa-miR-582-5p	−2.45	0.001
hsa-miR-424-3p	−2.49	0.001
hsa-miR-545-3p	−2.75	0.002
hsa-miR-548d-3p	−2.76	0.010

DEmiRNAs, differentially expressed miRNAs, CPP, central precocious puberty; ET, exaggerated thelarche.

## Data Availability

The original contributions presented in this study are included in the article. Further inquiries can be directed to the corresponding author(s). However, individual patient data protected by confidentiality and ethical regulations will not be shared.

## Supplementary Materials

The following supporting information can be downloaded at: https://www.mdpi.com/article/10.3390/ijms27041742/s1, Figure S1: Heatmap of exosomal miRNA profiles in the CPP, ET, PT and CNT groups; Table S1: List of Differentially Expressed miRNAs in Central Precocious Puberty vs. Controls; Table S2: List of Differentially Expressed miRNAs in Exaggerated Thelarche vs. Controls; Table S3: List of Differentially Expressed miRNAs in Premature Thelarche vs. Controls.

## Abbreviations

The following abbreviations are used in this manuscript:

AGE–RAGE	Advanced glycation end product (AGE)–receptor of AGE
BA	Bone age
BMI	Body mass index
CA	Chronological age
cDNA	Complementary DNA
CNT	Control
CPP	Central precocious puberty
DLK1	delta-like noncanonical Notch ligand 1
DNA	Deoxyribonucleic acid
E2	EstradiolfoxO
FC	Fold change
FoxO	Forkhead box
FSH	Follicle-stimulating hormone
GnRH	Gonadotropin-releasing hormone
HIF-1	Hypoxia-inducible factor 1
HPG	Hypothalamic–pituitary–gonadal
IQR	Interquartile range
KEGG	Kyoto Encyclopedia of Genes and Genomes
LH	Luteinizing hormone
MAPK	Mitogen-activated protein kinase
mTOR	Mammalian target of rapamycin
miRNA	micro ribonucleic acid
RPM	reads per million mapped reads
SDS	standard deviation score
