# Supplementary material for: Differential microRNA Expression Profiles in Girls with Idiopathic Central Precocious Puberty and Premature Thelarche
Source: Int J Mol Sci. 2026 Feb 11;27(4):1742. doi: 10.3390/ijms27041742 (PMC12941009; doi:10.3390/ijms27041742)
Supplement: Supplementary file 1 [file ijms-27-01742-s001.zip › ijms-4116126-supplementary.pdf]

Supplementary Materials

Differential mircoRNA Expression Profiles in Girls with Idiopathic Central Precocious Puberty and Premature Thelarche

Supplementary Figure S1. Heatmap of exosomal miRNA profiles in the CPP, ET, PT and CNT groups.

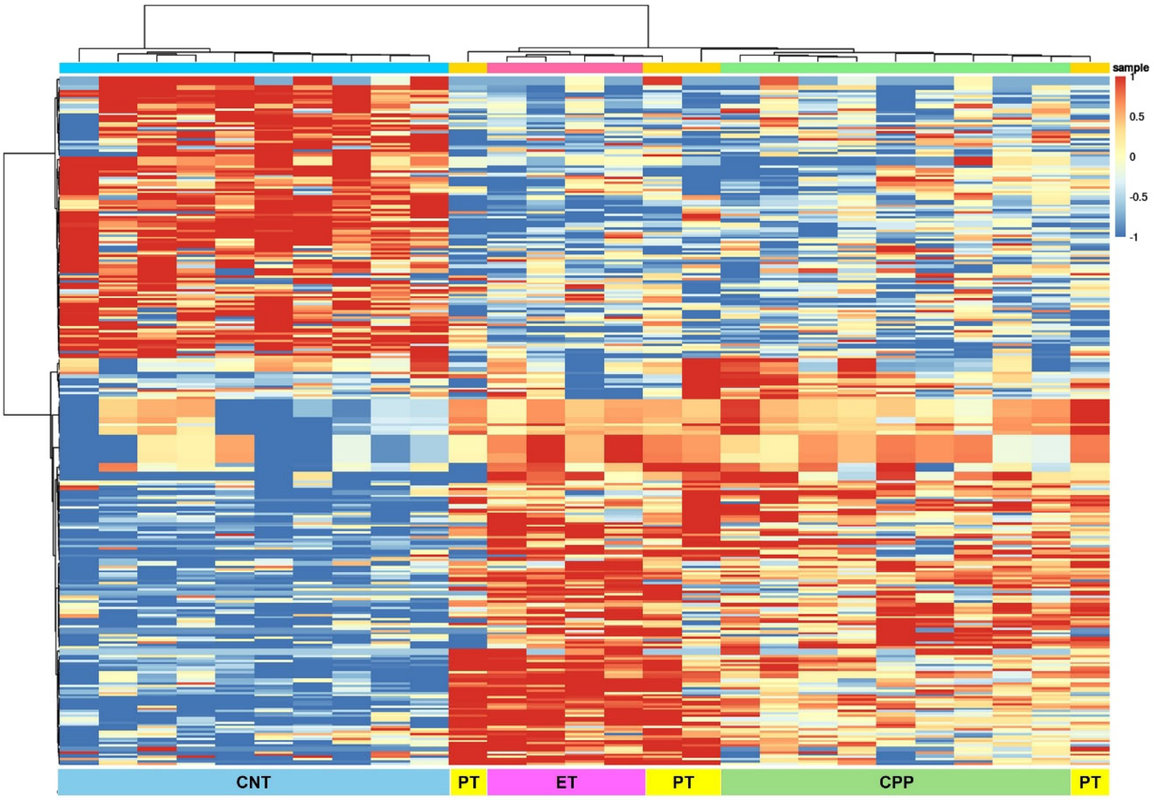

The miRNA expression profiles were distinct between the control group and all other groups. while the PT group did not clearly cluster separately from the ET and CPP groups.

CPP, central precocious puberty; ET, exaggerated thelarche; PT, premature thelarche; CNT, control.

Supplementary Table S1. List of Differentially Expressed miRNAs in Central Precocious Puberty vs. Controls

miRNA_precursor	log (fold change)	*P*-Value
hsa-miR-374a-5p_hsa-mir-374a	3.95	2.06E-24
hsa-miR-221-3p_hsa-mir-221	2.16	1.00E-22
hsa-miR-3605-3p_hsa-mir-3605	-2.50	3.21E-22
hsa-let-7d-3p_hsa-let-7d	-1.32	8.81E-21
hsa-miR-4429_hsa-mir-4429	3.28	1.99E-20
hsa-miR-22-3p_hsa-mir-22	2.00	4.83E-19

miRNA_precursor	log (fold change)	*P*-Value
hsa-miR-222-3p_hsa-mir-222	2.21	2.08E-18
hsa-miR-1224-5p_hsa-mir-1224	-2.74	1.09E-17
hsa-miR-20a-5p_hsa-mir-20a	2.78	2.49E-17
hsa-miR-760_hsa-mir-760	-2.14	3.47E-16
hsa-miR-374b-5p_hsa-mir-374b	3.78	7.57E-16
hsa-miR-193b-5p_hsa-mir-193b	-2.36	2.17E-15
hsa-miR-103a-3p_hsa-mir-103a-1	1.60	3.44E-15
hsa-miR-103a-3p_hsa-mir-103a-2	1.60	3.45E-15
hsa-miR-26b-5p_hsa-mir-26b	1.75	8.19E-15
hsa-miR-369-3p_hsa-mir-369	4.56	3.64E-14
hsa-miR-1468-5p_hsa-mir-1468	-3.15	1.89E-13
hsa-miR-129-5p_hsa-mir-129-1	-2.83	2.38E-13
hsa-miR-129-5p_hsa-mir-129-2	-2.83	2.38E-13
hsa-miR-3605-5p_hsa-mir-3605	-2.61	2.86E-12
hsa-miR-363-3p_hsa-mir-363	1.91	3.62E-12
hsa-miR-4732-5p_hsa-mir-4732	-2.06	1.28E-11
hsa-miR-30a-3p_hsa-mir-30a	-1.72	4.97E-11
hsa-miR-664b-5p_hsa-mir-664b	-2.25	6.77E-11
hsa-miR-423-3p_hsa-mir-423	1.85	7.54E-11
hsa-miR-181a-5p_hsa-mir-181a-2	1.79	8.42E-11
hsa-miR-181a-5p_hsa-mir-181a-1	1.79	8.45E-11
hsa-miR-4516_hsa-mir-4516	-3.61	1.11E-10
hsa-let-7b-5p_hsa-let-7b	-1.51	2.73E-10
hsa-miR-18a-5p_hsa-mir-18a	2.54	3.94E-10
hsa-let-7g-5p_hsa-let-7g	1.38	4.41E-10
hsa-miR-423-5p_hsa-mir-423	-1.14	5.74E-10
hsa-miR-107_hsa-mir-107	1.48	1.03E-09
hsa-miR-21-5p_hsa-mir-21	1.57	1.43E-09
hsa-miR-874-3p_hsa-mir-874	-2.02	1.74E-09
hsa-miR-17-3p_hsa-mir-17	3.76	2.40E-09
hsa-miR-451a_hsa-mir-451a	1.95	3.11E-09
hsa-miR-6734-5p_hsa-mir-6734	-1.90	3.63E-09
hsa-miR-590-3p_hsa-mir-590	2.47	3.74E-09
hsa-miR-142-3p_hsa-mir-142	2.04	3.98E-09
hsa-miR-335-5p_hsa-mir-335	1.63	6.05E-09
hsa-miR-1294_hsa-mir-1294	-1.62	7.40E-09
hsa-miR-125a-5p_hsa-mir-125a	-1.36	1.39E-08
hsa-miR-1277-5p_hsa-mir-1277	2.91	1.63E-08
hsa-miR-144-5p_hsa-mir-144	1.93	2.09E-08
hsa-miR-340-5p_hsa-mir-340	1.75	2.50E-08
hsa-miR-139-3p_hsa-mir-139	-1.81	2.96E-08
hsa-miR-15a-5p_hsa-mir-15a	1.35	3.56E-08
hsa-miR-628-5p_hsa-mir-628	3.05	3.78E-08
hsa-miR-4732-3p_hsa-mir-4732	-1.98	8.39E-08
hsa-miR-328-3p_hsa-mir-328	-1.21	9.25E-08
hsa-miR-98-5p_hsa-mir-98	1.55	1.46E-07
hsa-miR-125b-5p_hsa-mir-125b-2	-1.33	1.44E-07
hsa-miR-125b-5p_hsa-mir-125b-1	-1.33	1.50E-07

miRNA_precursor	log (fold change)	*P*-Value
hsa-miR-5187-5p_hsa-mir-5187	-1.71	2.19E-07
hsa-miR-548e-3p_hsa-mir-548e	4.25	4.53E-07
hsa-miR-17-5p_hsa-mir-17	1.50	4.59E-07
hsa-miR-379-5p_hsa-mir-379	2.28	7.90E-07
hsa-miR-424-5p_hsa-mir-424	1.30	8.14E-07
hsa-miR-381-3p_hsa-mir-381	2.03	1.36E-06
hsa-miR-22-5p_hsa-mir-22	2.89	1.63E-06
hsa-miR-19b-3p_hsa-mir-19b-1	1.48	1.83E-06
hsa-miR-19b-3p_hsa-mir-19b-2	1.47	1.80E-06
hsa-miR-122-5p_hsa-mir-122	-2.25	1.84E-06
hsa-miR-32-5p_hsa-mir-32	1.33	2.40E-06
hsa-miR-143-5p_hsa-mir-143	2.76	4.12E-06
hsa-miR-301a-3p_hsa-mir-301a	1.73	4.40E-06
hsa-miR-29b-3p_hsa-mir-29b-2	1.64	4.76E-06
hsa-miR-2116-3p_hsa-mir-2116	-2.15	4.76E-06
hsa-miR-29b-3p_hsa-mir-29b-1	1.64	4.84E-06
hsa-miR-16-2-3p_hsa-mir-16-2	1.56	5.21E-06
hsa-miR-15b-5p_hsa-mir-15b	1.17	6.46E-06
hsa-miR-144-3p_hsa-mir-144	1.70	6.82E-06
hsa-miR-1304-3p_hsa-mir-1304	-1.16	6.81E-06
hsa-miR-197-5p_hsa-mir-197	-1.81	6.88E-06
hsa-miR-6780a-5p_hsa-mir-6780a	-2.19	7.17E-06
hsa-miR-502-3p_hsa-mir-502	1.49	8.20E-06
hsa-miR-92b-3p_hsa-mir-92b	-1.31	9.32E-06
hsa-miR-454-3p_hsa-mir-454	1.40	1.06E-05
hsa-miR-4508_hsa-mir-4508	-1.46	1.15E-05
hsa-miR-493-5p_hsa-mir-493	2.55	1.17E-05
hsa-miR-19a-3p_hsa-mir-19a	1.66	1.29E-05
hsa-miR-378d_hsa-mir-378d-2	3.69	1.55E-05
hsa-miR-2355-3p_hsa-mir-2355	2.08	1.63E-05
hsa-miR-369-5p_hsa-mir-369	1.69	1.68E-05
hsa-miR-324-5p_hsa-mir-324	-1.00	1.73E-05
hsa-miR-652-3p_hsa-mir-652	1.32	1.84E-05
hsa-miR-33a-5p_hsa-mir-33a	2.65	2.01E-05
hsa-miR-24-3p_hsa-mir-24-2	1.02	1.99E-05
hsa-miR-483-5p_hsa-mir-483	-1.50	2.02E-05
hsa-miR-24-3p_hsa-mir-24-1	1.02	2.09E-05
hsa-miR-664a-5p_hsa-mir-664a	-1.04	2.09E-05
hsa-miR-28-5p_hsa-mir-28	2.84	2.48E-05
hsa-miR-142-5p_hsa-mir-142	1.39	2.48E-05
hsa-miR-615-3p_hsa-mir-615	-2.09	2.66E-05
hsa-miR-4750-5p_hsa-mir-4750	-2.40	2.68E-05
hsa-miR-654-3p_hsa-mir-654	1.84	3.04E-05
hsa-let-7b-3p_hsa-let-7b	-1.15	3.03E-05
hsa-miR-378d_hsa-mir-378d-1	3.43	3.20E-05
hsa-miR-1-3p_hsa-mir-1-1	1.74	4.94E-05
hsa-miR-20a-3p_hsa-mir-20a	3.67	5.35E-05
hsa-miR-1-3p_hsa-mir-1-2	1.55	5.76E-05

miRNA_precursor	log (fold change)	*P*-Value
hsa-miR-5010-5p_hsa-mir-5010	-2.02	5.71E-05
hsa-miR-4433a-3p_hsa-mir-4433a	2.42	6.42E-05
hsa-miR-150-3p_hsa-mir-150	-1.48	6.87E-05
hsa-miR-3960_hsa-mir-3960	-2.11	6.87E-05
hsa-miR-190a-5p_hsa-mir-190a	1.50	7.05E-05
hsa-miR-1290_hsa-mir-1290	-1.46	7.57E-05
hsa-miR-3187-3p_hsa-mir-3187	-1.35	8.42E-05
hsa-miR-151a-3p_hsa-mir-151a	-1.10	9.09E-05
hsa-miR-146b-5p_hsa-mir-146b	1.17	1.02E-04
hsa-miR-576-3p_hsa-mir-576	2.96	1.05E-04
hsa-miR-548ad-5p_hsa-mir-548ae-2	1.77	1.10E-04
hsa-miR-548ae-5p_hsa-mir-548ae-2	1.77	1.10E-04
hsa-miR-1226-3p_hsa-mir-1226	-2.52	1.09E-04
hsa-miR-941_hsa-mir-941-1	-1.34	1.20E-04
hsa-miR-941_hsa-mir-941-2	-1.34	1.20E-04
hsa-miR-941_hsa-mir-941-3	-1.34	1.20E-04
hsa-miR-941_hsa-mir-941-4	-1.34	1.20E-04
hsa-miR-941_hsa-mir-941-5	-1.34	1.20E-04
hsa-miR-196a-5p_hsa-mir-196a-2	-1.54	1.19E-04
hsa-miR-31-5p_hsa-mir-31	2.43	1.28E-04
hsa-miR-143-3p_hsa-mir-143	1.50	1.32E-04
hsa-miR-503-5p_hsa-mir-503	-1.19	1.35E-04
hsa-miR-99b-5p_hsa-mir-99b	-1.24	1.64E-04
hsa-miR-196a-5p_hsa-mir-196a-1	-1.44	1.85E-04
hsa-miR-505-5p_hsa-mir-505	-1.48	1.98E-04
hsa-miR-483-3p_hsa-mir-483	-1.60	2.00E-04
hsa-miR-877-5p_hsa-mir-877	-1.61	2.16E-04
hsa-miR-377-3p_hsa-mir-377	2.10	2.30E-04
hsa-miR-125b-2-3p_hsa-mir-125b-2	1.68	2.38E-04
hsa-miR-574-3p_hsa-mir-574	-1.07	2.45E-04
hsa-miR-365a-5p_hsa-mir-365a	-3.02	2.62E-04
hsa-miR-4738-3p_hsa-mir-4738	-2.18	2.89E-04
hsa-miR-376a-3p_hsa-mir-376a-1	1.71	2.97E-04
hsa-miR-376a-3p_hsa-mir-376a-2	1.71	2.97E-04
hsa-miR-6731-5p_hsa-mir-6731	-2.24	2.99E-04
hsa-miR-6767-5p_hsa-mir-6767	-2.19	3.18E-04
hsa-miR-6779-5p_hsa-mir-6779	-3.08	3.40E-04
hsa-miR-542-3p_hsa-mir-542	1.33	3.67E-04
hsa-miR-885-3p_hsa-mir-885	-2.23	3.83E-04
hsa-miR-636_hsa-mir-636	-1.53	3.87E-04
hsa-miR-548l_hsa-mir-548l	2.16	3.97E-04
hsa-miR-106b-5p_hsa-mir-106b	1.78	4.12E-04
hsa-miR-7706_hsa-mir-7706	-1.68	4.12E-04
hsa-miR-3913-5p_hsa-mir-3913-1	-1.52	4.67E-04
hsa-miR-3913-5p_hsa-mir-3913-2	-1.52	4.67E-04
hsa-miR-6891-5p_hsa-mir-6891	-2.52	4.68E-04
hsa-miR-432-5p_hsa-mir-432	-1.21	4.78E-04
hsa-miR-181c-5p_hsa-mir-181c	1.83	5.15E-04

miRNA_precursor	log (fold change)	*P*-Value
hsa-miR-338-3p_hsa-mir-338	1.11	5.17E-04
hsa-miR-3120-3p_hsa-mir-3120	2.79	6.13E-04
hsa-miR-6807-5p_hsa-mir-6807	-2.06	6.10E-04
hsa-miR-548ad-5p_hsa-mir-548ad	1.63	6.28E-04
hsa-miR-548ae-5p_hsa-mir-548ad	1.63	6.28E-04
hsa-miR-450b-5p_hsa-mir-450b	1.43	6.85E-04
hsa-miR-376c-3p_hsa-mir-376c	1.96	7.09E-04
hsa-miR-651-5p_hsa-mir-651	1.38	7.44E-04
hsa-miR-7976_hsa-mir-7976	-1.39	8.57E-04
hsa-miR-494-3p_hsa-mir-494	1.57	9.10E-04
hsa-miR-4433a-5p_hsa-mir-4433a	-2.03	9.83E-04
hsa-miR-431-5p_hsa-mir-431	1.43	9.89E-04
hsa-miR-4669_hsa-mir-4669	-2.48	9.97E-04
hsa-miR-191-3p_hsa-mir-191	-1.03	1.07E-03
hsa-miR-7704_hsa-mir-7704	-1.82	1.15E-03
hsa-miR-6511b-3p_hsa-mir-6511b-1	-2.11	1.23E-03
hsa-miR-181d-5p_hsa-mir-181d	1.59	1.26E-03
hsa-miR-493-3p_hsa-mir-493	1.82	1.28E-03
hsa-miR-6511a-3p_hsa-mir-6511a-1	-1.58	1.31E-03
hsa-miR-6511a-3p_hsa-mir-6511a-2	-1.58	1.31E-03
hsa-miR-6511a-3p_hsa-mir-6511a-3	-1.58	1.31E-03
hsa-miR-6511a-3p_hsa-mir-6511a-4	-1.58	1.31E-03
hsa-miR-548ad-5p_hsa-mir-548ay	1.51	1.36E-03
hsa-miR-548ae-5p_hsa-mir-548ay	1.51	1.36E-03
hsa-miR-548ay-5p_hsa-mir-548ay	1.51	1.36E-03
hsa-miR-548ad-5p_hsa-mir-548d-1	1.41	1.42E-03
hsa-miR-548ad-5p_hsa-mir-548d-2	1.41	1.42E-03
hsa-miR-548ae-5p_hsa-mir-548d-1	1.41	1.42E-03
hsa-miR-548ae-5p_hsa-mir-548d-2	1.41	1.42E-03
hsa-miR-548ay-5p_hsa-mir-548d-1	1.41	1.42E-03
hsa-miR-548ay-5p_hsa-mir-548d-2	1.41	1.42E-03
hsa-miR-548d-5p_hsa-mir-548d-1	1.41	1.42E-03
hsa-miR-548d-5p_hsa-mir-548d-2	1.41	1.42E-03
hsa-miR-6741-5p_hsa-mir-6741	-2.00	1.44E-03
hsa-miR-378c_hsa-mir-378c	1.17	1.53E-03
hsa-miR-3691-5p_hsa-mir-3691	-2.31	1.54E-03
hsa-miR-125a-3p_hsa-mir-125a	-1.32	1.62E-03
hsa-miR-1236-5p_hsa-mir-1236	-2.61	1.65E-03
hsa-miR-6859-3p_hsa-mir-6859-1	-2.63	1.68E-03
hsa-miR-6859-3p_hsa-mir-6859-2	-2.63	1.68E-03
hsa-miR-6859-3p_hsa-mir-6859-3	-2.63	1.68E-03
hsa-miR-6859-3p_hsa-mir-6859-4	-2.63	1.68E-03
hsa-miR-3154_hsa-mir-3154	-1.75	1.71E-03
hsa-miR-6511b-3p_hsa-mir-6511b-2	-2.04	2.38E-03
hsa-miR-4446-3p_hsa-mir-4446	-1.53	2.55E-03
hsa-miR-375-3p_hsa-mir-375	-1.15	2.65E-03
hsa-miR-181c-3p_hsa-mir-181c	1.40	2.73E-03
hsa-miR-18a-3p_hsa-mir-18a	1.78	2.81E-03

miRNA_precursor	log (fold change)	*P*-Value
hsa-miR-1908-5p_hsa-mir-1908	-1.18	2.81E-03
hsa-miR-5196-3p_hsa-mir-5196	-2.28	2.90E-03
hsa-miR-548am-5p_hsa-mir-548c	1.67	3.13E-03
hsa-miR-548am-5p_hsa-mir-548o-2	1.67	3.13E-03
hsa-miR-548au-5p_hsa-mir-548c	1.67	3.13E-03
hsa-miR-548au-5p_hsa-mir-548o-2	1.67	3.13E-03
hsa-miR-548c-5p_hsa-mir-548c	1.67	3.13E-03
hsa-miR-548c-5p_hsa-mir-548o-2	1.67	3.13E-03
hsa-miR-548o-5p_hsa-mir-548c	1.67	3.13E-03
hsa-miR-548o-5p_hsa-mir-548o-2	1.67	3.13E-03
hsa-miR-1250-5p_hsa-mir-1250	1.67	3.04E-03
hsa-miR-206_hsa-mir-206	-1.88	3.16E-03
hsa-miR-548a-3p_hsa-mir-548a-1	1.81	3.24E-03
hsa-miR-548a-3p_hsa-mir-548a-2	1.81	3.24E-03
hsa-miR-548a-3p_hsa-mir-548a-3	1.81	3.24E-03
hsa-miR-4766-3p_hsa-mir-4766	2.36	3.53E-03
hsa-miR-140-5p_hsa-mir-140	1.05	3.53E-03
hsa-miR-7-1-3p_hsa-mir-7-1	1.91	3.63E-03
hsa-miR-3124-5p_hsa-mir-3124	-1.80	3.66E-03
hsa-miR-337-5p_hsa-mir-337	1.27	3.74E-03
hsa-miR-1306-5p_hsa-mir-1306	-1.16	3.74E-03
hsa-miR-548am-5p_hsa-mir-548am	1.64	3.88E-03
hsa-miR-548au-5p_hsa-mir-548am	1.64	3.88E-03
hsa-miR-548c-5p_hsa-mir-548am	1.64	3.88E-03
hsa-miR-548o-5p_hsa-mir-548am	1.64	3.88E-03
hsa-miR-132-5p_hsa-mir-132	2.10	3.91E-03
hsa-miR-429_hsa-mir-429	1.54	4.01E-03
hsa-miR-186-3p_hsa-mir-186	2.31	4.06E-03
hsa-miR-550a-3-5p_hsa-mir-550a-1	2.55	4.35E-03
hsa-miR-550a-3-5p_hsa-mir-550a-2	2.55	4.35E-03
hsa-miR-550a-5p_hsa-mir-550a-1	2.55	4.35E-03
hsa-miR-550a-5p_hsa-mir-550a-2	2.55	4.35E-03
hsa-miR-1299_hsa-mir-1299	-2.10	4.38E-03
hsa-miR-18b-5p_hsa-mir-18b	2.16	4.57E-03
hsa-miR-6782-3p_hsa-mir-6782	-2.35	4.61E-03
hsa-miR-29c-5p_hsa-mir-29c	1.23	4.70E-03
hsa-miR-1538_hsa-mir-1538	-1.96	5.03E-03
hsa-miR-378i_hsa-mir-378i	1.31	5.09E-03
hsa-miR-885-5p_hsa-mir-885	-1.69	5.22E-03
hsa-miR-202-3p_hsa-mir-202	-1.57	5.52E-03
hsa-miR-3940-3p_hsa-mir-3940	-1.70	5.82E-03
hsa-miR-30c-2-3p_hsa-mir-30c-2	-1.89	5.85E-03
hsa-miR-96-5p_hsa-mir-96	1.22	6.54E-03
hsa-miR-6514-5p_hsa-mir-6514	-1.89	6.62E-03
hsa-miR-3131_hsa-mir-3131	-2.13	7.06E-03
hsa-miR-204-5p_hsa-mir-204	-1.46	7.17E-03
hsa-miR-1273h-5p_hsa-mir-1273h	-2.02	7.18E-03
hsa-miR-320e_hsa-mir-320e	1.05	7.27E-03

miRNA_precursor	log (fold change)	*P*-Value
hsa-miR-4489_hsa-mir-4489	-2.20	7.74E-03
hsa-miR-215-5p_hsa-mir-215	1.12	7.92E-03
hsa-miR-1976_hsa-mir-1976	-1.88	8.32E-03
hsa-miR-362-5p_hsa-mir-362	1.90	8.57E-03
hsa-miR-6805-5p_hsa-mir-6805	-1.15	9.44E-03
hsa-miR-668-3p_hsa-mir-668	-1.71	9.43E-03
hsa-miR-374a-3p_hsa-mir-374a	1.37	9.70E-03
hsa-miR-3198_hsa-mir-3198-1	-1.57	0.01
hsa-miR-3198_hsa-mir-3198-2	-1.57	0.01
hsa-miR-6724-5p_hsa-mir-6724-1	-1.69	0.01
hsa-miR-6724-5p_hsa-mir-6724-2	-1.69	0.01
hsa-miR-6724-5p_hsa-mir-6724-3	-1.69	0.01
hsa-miR-6724-5p_hsa-mir-6724-4	-1.69	0.01
hsa-miR-539-3p_hsa-mir-539	2.17	0.01
hsa-miR-487b-3p_hsa-mir-487b	1.45	0.01
hsa-miR-1255b-5p_hsa-mir-1255b-1	-1.25	0.01
hsa-miR-610_hsa-mir-610	-2.01	0.01
hsa-miR-1255b-5p_hsa-mir-1255b-2	-1.24	0.01
hsa-miR-411-5p_hsa-mir-411	1.67	0.01
hsa-miR-3065-3p_hsa-mir-3065	2.07	0.01
hsa-miR-6842-5p_hsa-mir-6842	-1.91	0.01
hsa-miR-9-3p_hsa-mir-9-1	1.25	0.01
hsa-miR-9-3p_hsa-mir-9-2	1.25	0.01
hsa-miR-9-3p_hsa-mir-9-3	1.25	0.01
hsa-miR-3173-5p_hsa-mir-3173	-1.02	0.01
hsa-miR-4511_hsa-mir-4511	-1.51	0.01
hsa-miR-551b-3p_hsa-mir-551b	2.24	0.02
hsa-miR-193b-3p_hsa-mir-193b	1.74	0.02
hsa-miR-221-5p_hsa-mir-221	1.30	0.02
hsa-miR-23a-5p_hsa-mir-23a	-1.04	0.02
hsa-miR-211-5p_hsa-mir-211	-1.69	0.02
hsa-miR-500a-5p_hsa-mir-500a	1.91	0.02
hsa-miR-500a-5p_hsa-mir-500b	1.91	0.02
hsa-miR-500b-5p_hsa-mir-500a	1.91	0.02
hsa-miR-500b-5p_hsa-mir-500b	1.91	0.02
hsa-miR-146b-3p_hsa-mir-146b	-1.01	0.02
hsa-miR-11401_hsa-mir-11401	-1.32	0.02
hsa-miR-1247-5p_hsa-mir-1247	-1.20	0.02
hsa-miR-4665-5p_hsa-mir-4665	-1.80	0.02
hsa-miR-504-5p_hsa-mir-504	-1.75	0.02
hsa-miR-3939_hsa-mir-3939	-1.92	0.02
hsa-miR-543_hsa-mir-543	1.99	0.02
hsa-miR-3127-5p_hsa-mir-3127	-1.20	0.02
hsa-miR-7977_hsa-mir-7977	1.52	0.02
hsa-miR-6793-5p_hsa-mir-6793	-1.44	0.02
hsa-miR-10401-3p_hsa-mir-10401	-1.05	0.02
hsa-miR-1270_hsa-mir-1270	-1.02	0.03
hsa-miR-433-3p_hsa-mir-433	-1.57	0.03

miRNA_precursor	log (fold change)	*P*-Value
hsa-miR-329-3p_hsa-mir-329-1	1.46	0.03
hsa-miR-329-3p_hsa-mir-329-2	1.46	0.03
hsa-miR-1343-3p_hsa-mir-1343	-1.33	0.03
hsa-miR-146a-3p_hsa-mir-146a	1.41	0.03
hsa-miR-1179_hsa-mir-1179	1.51	0.03
hsa-miR-548au-5p_hsa-mir-548au	1.26	0.03
hsa-miR-501-5p_hsa-mir-501	1.75	0.03
hsa-miR-3158-3p_hsa-mir-3158-1	1.35	0.03
hsa-miR-3158-3p_hsa-mir-3158-2	1.35	0.03
hsa-miR-148a-5p_hsa-mir-148a	1.23	0.03
hsa-miR-3064-5p_hsa-mir-3064	-1.43	0.04
hsa-miR-3942-5p_hsa-mir-3942	1.76	0.04
hsa-miR-1185-1-3p_hsa-mir-1185-1	1.42	0.04
hsa-miR-548at-5p_hsa-mir-548at	1.62	0.04
hsa-miR-34c-3p_hsa-mir-34c	-1.87	0.04
hsa-miR-409-5p_hsa-mir-409	1.74	0.04
hsa-miR-30c-1-3p_hsa-mir-30c-1	-1.49	0.04
hsa-miR-98-3p_hsa-mir-98	1.85	0.04
hsa-miR-187-3p_hsa-mir-187	1.82	0.04
hsa-miR-133a-3p_hsa-mir-133a-1	1.10	0.045
hsa-miR-133a-3p_hsa-mir-133a-2	1.10	0.045
hsa-miR-551a_hsa-mir-551a	1.41	0.047
hsa-miR-3143_hsa-mir-3143	1.23	0.048
hsa-miR-4454_hsa-mir-4454	1.41	0.048

Supplementary Table S2. List of Differentially Expressed miRNAs in Exaggerated Thelarche vs. Controls

miRNA_precursor	log (fold change)	*P*-Value
hsa-miR-22-3p_hsa-mir-22	3.15	5.90E-47
hsa-miR-4429_hsa-mir-4429	4.06	1.25E-29
hsa-miR-374a-5p_hsa-mir-374a	4.41	4.57E-24
hsa-miR-222-3p_hsa-mir-222	2.84	9.90E-22
hsa-miR-221-3p_hsa-mir-221	2.33	1.49E-19
hsa-miR-20a-5p_hsa-mir-20a	3.65	1.36E-18
hsa-miR-18a-5p_hsa-mir-18a	4.10	2.45E-17
hsa-miR-1224-5p_hsa-mir-1224	-3.50	1.39E-16
hsa-miR-139-3p_hsa-mir-139	-2.72	2.01E-16
hsa-miR-15b-5p_hsa-mir-15b	2.12	2.99E-16
hsa-miR-424-5p_hsa-mir-424	2.84	7.08E-16
hsa-let-7g-5p_hsa-let-7g	2.27	1.99E-15
hsa-miR-15a-5p_hsa-mir-15a	2.14	4.55E-15
hsa-miR-17-5p_hsa-mir-17	2.88	1.21E-14
hsa-miR-374b-5p_hsa-mir-374b	3.98	6.89E-14

**miRNA_precursor**	**log (fold change)**	***P*-Value**
hsa-miR-107_hsa-mir-107	1.64	2.49E-13
hsa-miR-144-3p_hsa-mir-144	3.21	3.58E-13
hsa-miR-760_hsa-mir-760	-2.94	1.10E-12
hsa-miR-451a_hsa-mir-451a	2.59	1.80E-12
hsa-miR-24-3p_hsa-mir-24-2	2.27	2.17E-12
hsa-miR-24-3p_hsa-mir-24-1	2.27	2.35E-12
hsa-miR-27a-3p_hsa-mir-27a	1.81	8.71E-12
hsa-miR-30b-5p_hsa-mir-30b	2.42	1.24E-10
hsa-miR-1294_hsa-mir-1294	-2.29	1.80E-10
hsa-miR-26b-5p_hsa-mir-26b	1.60	1.85E-10
hsa-miR-106b-5p_hsa-mir-106b	3.42	2.33E-10
hsa-miR-98-5p_hsa-mir-98	1.79	5.02E-10
hsa-miR-7-1-3p_hsa-mir-7-1	3.84	6.04E-10
hsa-miR-4508_hsa-mir-4508	-2.00	9.59E-10
hsa-miR-129-5p_hsa-mir-129-1	-3.41	1.31E-09
hsa-miR-129-5p_hsa-mir-129-2	-3.41	1.31E-09
hsa-miR-423-3p_hsa-mir-423	2.19	2.00E-09
hsa-miR-16-2-3p_hsa-mir-16-2	2.37	2.41E-09
hsa-miR-199a-5p_hsa-mir-199a-2	2.10	3.62E-09
hsa-miR-199a-5p_hsa-mir-199a-1	2.10	4.17E-09
hsa-miR-301a-3p_hsa-mir-301a	2.75	5.57E-09
hsa-miR-142-3p_hsa-mir-142	2.14	1.26E-08
hsa-miR-125a-5p_hsa-mir-125a	-2.06	1.69E-08
hsa-miR-22-5p_hsa-mir-22	3.62	1.95E-08
hsa-miR-1277-5p_hsa-mir-1277	2.38	2.07E-08
hsa-miR-19b-3p_hsa-mir-19b-1	2.17	2.62E-08
hsa-miR-19b-3p_hsa-mir-19b-2	2.17	2.63E-08
hsa-miR-3605-3p_hsa-mir-3605	-2.09	3.32E-08
hsa-miR-30a-3p_hsa-mir-30a	-2.03	4.99E-08
hsa-let-7b-5p_hsa-let-7b	-1.89	9.59E-08
hsa-miR-4732-5p_hsa-mir-4732	-1.96	9.86E-08
hsa-miR-664a-5p_hsa-mir-664a	-2.02	1.12E-07
hsa-miR-664b-5p_hsa-mir-664b	-2.91	1.65E-07
hsa-miR-30c-5p_hsa-mir-30c-2	1.61	1.72E-07
hsa-miR-30c-5p_hsa-mir-30c-1	1.61	1.74E-07
hsa-miR-625-5p_hsa-mir-625	2.19	2.07E-07
hsa-miR-23a-3p_hsa-mir-23a	1.61	2.84E-07
hsa-miR-320a-3p_hsa-mir-320a	1.49	4.24E-07
hsa-miR-125b-5p_hsa-mir-125b-2	-1.86	6.36E-07

miRNA_precursor	log (fold change)	*P*-Value
hsa-miR-125b-5p_hsa-mir-125b-1	-1.86	6.52E-07
hsa-miR-5187-5p_hsa-mir-5187	-2.36	6.55E-07
hsa-miR-337-5p_hsa-mir-337	2.42	7.59E-07
hsa-miR-375-3p_hsa-mir-375	-2.65	7.64E-07
hsa-miR-652-3p_hsa-mir-652	2.00	8.34E-07
hsa-miR-28-5p_hsa-mir-28	3.24	1.00E-06
hsa-miR-151a-3p_hsa-mir-151a	-1.81	1.14E-06
hsa-miR-484_hsa-mir-484	1.67	1.82E-06
hsa-miR-4286_hsa-mir-4286	2.36	1.98E-06
hsa-miR-590-3p_hsa-mir-590	2.62	2.54E-06
hsa-miR-19a-3p_hsa-mir-19a	2.19	3.24E-06
hsa-miR-4433a-3p_hsa-mir-4433a	3.44	4.26E-06
hsa-miR-502-3p_hsa-mir-502	1.65	4.50E-06
hsa-miR-29b-3p_hsa-mir-29b-2	2.20	4.59E-06
hsa-miR-4516_hsa-mir-4516	-3.98	4.66E-06
hsa-miR-29b-3p_hsa-mir-29b-1	2.19	4.67E-06
hsa-miR-1307-5p_hsa-mir-1307	1.66	5.02E-06
hsa-miR-339-3p_hsa-mir-339	1.46	5.02E-06
hsa-miR-23b-3p_hsa-mir-23b	1.49	5.15E-06
hsa-miR-103a-3p_hsa-mir-103a-1	1.10	5.21E-06
hsa-miR-103a-3p_hsa-mir-103a-2	1.10	5.28E-06
hsa-miR-1304-3p_hsa-mir-1304	-1.64	8.67E-06
hsa-miR-369-3p_hsa-mir-369	3.31	1.17E-05
hsa-miR-454-3p_hsa-mir-454	1.86	1.35E-05
hsa-miR-3187-3p_hsa-mir-3187	-2.19	1.53E-05
hsa-miR-335-5p_hsa-mir-335	1.46	1.64E-05
hsa-miR-148b-3p_hsa-mir-148b	1.10	1.69E-05
hsa-miR-186-5p_hsa-mir-186	1.50	1.69E-05
hsa-miR-130a-3p_hsa-mir-130a	1.27	2.03E-05
hsa-miR-1290_hsa-mir-1290	-2.55	2.13E-05
hsa-miR-92b-3p_hsa-mir-92b	-1.76	2.40E-05
hsa-miR-338-3p_hsa-mir-338	1.48	2.55E-05
hsa-miR-1246_hsa-mir-1246	-2.70	2.63E-05
hsa-miR-206_hsa-mir-206	-3.29	4.23E-05
hsa-miR-196a-5p_hsa-mir-196a-1	-2.19	4.34E-05
hsa-miR-2355-3p_hsa-mir-2355	2.31	4.77E-05
hsa-miR-4732-3p_hsa-mir-4732	-1.80	5.06E-05
hsa-miR-29c-5p_hsa-mir-29c	2.18	5.24E-05
hsa-miR-30d-5p_hsa-mir-30d	-1.24	5.72E-05

miRNA_precursor	log (fold change)	*P*-Value
hsa-miR-328-3p_hsa-mir-328	-1.34	5.94E-05
hsa-miR-196a-5p_hsa-mir-196a-2	-2.20	6.44E-05
hsa-miR-548am-5p_hsa-mir-548c	2.48	7.44E-05
hsa-miR-548am-5p_hsa-mir-548o-2	2.48	7.44E-05
hsa-miR-548au-5p_hsa-mir-548c	2.48	7.44E-05
hsa-miR-548au-5p_hsa-mir-548o-2	2.48	7.44E-05
hsa-miR-548c-5p_hsa-mir-548c	2.48	7.44E-05
hsa-miR-548c-5p_hsa-mir-548o-2	2.48	7.44E-05
hsa-miR-548o-5p_hsa-mir-548c	2.48	7.44E-05
hsa-miR-548o-5p_hsa-mir-548o-2	2.48	7.44E-05
hsa-miR-340-5p_hsa-mir-340	1.28	7.96E-05
hsa-miR-6805-5p_hsa-mir-6805	-2.93	8.18E-05
hsa-miR-99b-5p_hsa-mir-99b	-2.00	9.39E-05
hsa-miR-532-5p_hsa-mir-532	-1.38	9.63E-05
hsa-miR-7704_hsa-mir-7704	-3.63	1.01E-04
hsa-miR-193b-5p_hsa-mir-193b	-1.86	1.12E-04
hsa-miR-140-5p_hsa-mir-140	1.66	1.14E-04
hsa-miR-548am-5p_hsa-mir-548am	2.42	1.18E-04
hsa-miR-548au-5p_hsa-mir-548am	2.42	1.18E-04
hsa-miR-548c-5p_hsa-mir-548am	2.42	1.18E-04
hsa-miR-548o-5p_hsa-mir-548am	2.42	1.18E-04
hsa-miR-143-5p_hsa-mir-143	2.62	1.21E-04
hsa-miR-339-5p_hsa-mir-339	1.14	1.61E-04
hsa-miR-6734-5p_hsa-mir-6734	-1.59	1.66E-04
hsa-miR-320e_hsa-mir-320e	1.73	1.68E-04
hsa-miR-3154_hsa-mir-3154	-3.21	1.72E-04
hsa-miR-432-5p_hsa-mir-432	-2.09	2.04E-04
hsa-miR-494-3p_hsa-mir-494	1.96	2.26E-04
hsa-miR-1299_hsa-mir-1299	-4.32	2.27E-04
hsa-miR-625-3p_hsa-mir-625	-1.25	2.32E-04
hsa-miR-499a-5p_hsa-mir-499a	2.61	2.39E-04
hsa-let-7c-5p_hsa-let-7c	-1.06	2.44E-04
hsa-let-7i-5p_hsa-let-7i	-1.00	2.61E-04
hsa-miR-627-5p_hsa-mir-627	2.24	2.80E-04
hsa-miR-486-5p_hsa-mir-486-1	-1.37	2.84E-04
hsa-miR-486-5p_hsa-mir-486-2	-1.37	2.86E-04
hsa-miR-30a-5p_hsa-mir-30a	-1.17	3.68E-04
hsa-miR-32-5p_hsa-mir-32	1.18	3.85E-04
hsa-miR-28-3p_hsa-mir-28	-1.43	3.87E-04

miRNA_precursor	log (fold change)	*P*-Value
hsa-miR-4750-5p_hsa-mir-4750	-2.80	4.21E-04
hsa-miR-6807-5p_hsa-mir-6807	-3.52	4.39E-04
hsa-miR-142-5p_hsa-mir-142	1.57	4.54E-04
hsa-miR-3127-5p_hsa-mir-3127	-2.78	4.71E-04
hsa-miR-363-3p_hsa-mir-363	1.04	4.75E-04
hsa-miR-483-3p_hsa-mir-483	-1.96	4.82E-04
hsa-miR-628-5p_hsa-mir-628	2.31	4.88E-04
hsa-miR-4511_hsa-mir-4511	-3.41	5.19E-04
hsa-miR-6731-5p_hsa-mir-6731	-3.11	5.78E-04
hsa-miR-1185-1-3p_hsa-mir-1185-1	2.61	5.89E-04
hsa-miR-376c-3p_hsa-mir-376c	2.38	5.98E-04
hsa-miR-133a-3p_hsa-mir-133a-1	1.81	8.26E-04
hsa-miR-133a-3p_hsa-mir-133a-2	1.81	8.26E-04
hsa-miR-197-5p_hsa-mir-197	-1.79	9.69E-04
hsa-miR-2116-3p_hsa-mir-2116	-1.96	1.01E-03
hsa-miR-1260a_hsa-mir-1260a	1.39	1.06E-03
hsa-miR-223-3p_hsa-mir-223	1.21	1.16E-03
hsa-miR-376a-3p_hsa-mir-376a-1	1.79	1.25E-03
hsa-miR-376a-3p_hsa-mir-376a-2	1.79	1.25E-03
hsa-miR-1468-5p_hsa-mir-1468	-1.69	1.27E-03
hsa-miR-378d_hsa-mir-378d-1	3.14	1.27E-03
hsa-miR-18a-3p_hsa-mir-18a	2.02	1.28E-03
hsa-miR-941_hsa-mir-941-1	-1.71	1.29E-03
hsa-miR-941_hsa-mir-941-2	-1.71	1.29E-03
hsa-miR-941_hsa-mir-941-3	-1.71	1.29E-03
hsa-miR-941_hsa-mir-941-4	-1.71	1.29E-03
hsa-miR-941_hsa-mir-941-5	-1.71	1.29E-03
hsa-miR-3120-3p_hsa-mir-3120	2.81	1.39E-03
hsa-miR-3605-5p_hsa-mir-3605	-1.38	1.48E-03
hsa-miR-15b-3p_hsa-mir-15b	1.42	1.84E-03
hsa-miR-483-5p_hsa-mir-483	-1.88	1.86E-03
hsa-miR-3150b-3p_hsa-mir-3150b	-2.46	2.04E-03
hsa-miR-31-5p_hsa-mir-31	2.31	2.17E-03
hsa-let-7i-3p_hsa-let-7i	1.57	2.28E-03
hsa-miR-1255b-5p_hsa-mir-1255b-1	-1.85	2.30E-03
hsa-miR-1255b-5p_hsa-mir-1255b-2	-1.84	2.35E-03
hsa-miR-1275_hsa-mir-1275	-3.19	2.42E-03
hsa-let-7g-3p_hsa-let-7g	1.85	2.44E-03
hsa-miR-202-3p_hsa-mir-202	-2.38	2.51E-03

miRNA_precursor	log (fold change)	*P*-Value
hsa-miR-3198_hsa-mir-3198-1	-2.84	2.89E-03
hsa-miR-3198_hsa-mir-3198-2	-2.84	2.89E-03
hsa-miR-145-3p_hsa-mir-145	1.73	3.16E-03
hsa-miR-6767-5p_hsa-mir-6767	-2.30	3.30E-03
hsa-miR-326_hsa-mir-326	1.27	3.39E-03
hsa-miR-6793-5p_hsa-mir-6793	-2.96	3.39E-03
hsa-miR-6780a-5p_hsa-mir-6780a	-1.67	3.59E-03
hsa-miR-3913-5p_hsa-mir-3913-1	-1.63	3.83E-03
hsa-miR-3913-5p_hsa-mir-3913-2	-1.63	3.83E-03
hsa-miR-6511a-3p_hsa-mir-6511a-1	-1.70	3.88E-03
hsa-miR-6511a-3p_hsa-mir-6511a-2	-1.70	3.88E-03
hsa-miR-6511a-3p_hsa-mir-6511a-3	-1.70	3.88E-03
hsa-miR-6511a-3p_hsa-mir-6511a-4	-1.70	3.88E-03
hsa-miR-3960_hsa-mir-3960	-2.08	4.30E-03
hsa-miR-4433b-3p_hsa-mir-4433b	1.54	4.96E-03
hsa-miR-10a-3p_hsa-mir-10a	-1.30	5.00E-03
hsa-miR-1301-3p_hsa-mir-1301	1.03	5.02E-03
hsa-miR-183-5p_hsa-mir-183	-1.30	5.37E-03
hsa-miR-4738-3p_hsa-mir-4738	-2.37	5.38E-03
hsa-miR-30c-2-3p_hsa-mir-30c-2	-2.68	5.54E-03
hsa-miR-10a-5p_hsa-mir-10a	-1.26	5.87E-03
hsa-miR-377-3p_hsa-mir-377	1.90	6.07E-03
hsa-miR-6859-3p_hsa-mir-6859-1	-3.09	6.21E-03
hsa-miR-6859-3p_hsa-mir-6859-2	-3.09	6.21E-03
hsa-miR-6859-3p_hsa-mir-6859-3	-3.09	6.21E-03
hsa-miR-6859-3p_hsa-mir-6859-4	-3.09	6.21E-03
hsa-miR-127-3p_hsa-mir-127	-1.40	6.27E-03
hsa-miR-668-3p_hsa-mir-668	-2.64	6.40E-03
hsa-miR-1287-5p_hsa-mir-1287	-1.35	6.76E-03
hsa-miR-4467_hsa-mir-4467	-2.07	6.90E-03
hsa-miR-487b-3p_hsa-mir-487b	1.93	6.93E-03
hsa-miR-5196-3p_hsa-mir-5196	-2.88	7.09E-03
hsa-miR-296-5p_hsa-mir-296	1.41	7.41E-03
hsa-miR-101-5p_hsa-mir-101-1	1.85	7.42E-03
hsa-miR-3124-5p_hsa-mir-3124	-2.43	7.80E-03
hsa-miR-4433a-5p_hsa-mir-4433a	-2.32	7.81E-03
hsa-miR-182-5p_hsa-mir-182	-1.27	8.03E-03
hsa-miR-548ad-5p_hsa-mir-548ay	1.46	8.48E-03
hsa-miR-548ae-5p_hsa-mir-548ay	1.46	8.48E-03

miRNA_precursor	log (fold change)	*P*-Value
hsa-miR-548ay-5p_hsa-mir-548ay	1.46	8.48E-03
hsa-miR-1538_hsa-mir-1538	-2.73	8.50E-03
hsa-miR-1247-5p_hsa-mir-1247	-1.84	8.58E-03
hsa-miR-3131_hsa-mir-3131	-2.75	9.15E-03
hsa-miR-548au-5p_hsa-mir-548au	1.76	9.26E-03
hsa-miR-409-5p_hsa-mir-409	2.31	9.49E-03
hsa-miR-11401_hsa-mir-11401	-2.00	9.82E-03
hsa-miR-329-3p_hsa-mir-329-1	1.96	9.98E-03
hsa-miR-329-3p_hsa-mir-329-2	1.96	9.98E-03
hsa-miR-542-3p_hsa-mir-542	1.30	0.01
hsa-miR-4669_hsa-mir-4669	-2.01	0.01
hsa-miR-10b-5p_hsa-mir-10b	-1.39	0.01
hsa-miR-671-5p_hsa-mir-671	1.04	0.01
hsa-miR-493-5p_hsa-mir-493	1.97	0.01
hsa-miR-9-3p_hsa-mir-9-1	1.50	0.01
hsa-miR-9-3p_hsa-mir-9-2	1.50	0.01
hsa-miR-9-3p_hsa-mir-9-3	1.50	0.01
hsa-miR-92b-5p_hsa-mir-92b	-1.24	0.01
hsa-miR-500a-5p_hsa-mir-500a	2.24	0.01
hsa-miR-500a-5p_hsa-mir-500b	2.24	0.01
hsa-miR-7977_hsa-mir-7977	1.91	0.01
hsa-miR-379-5p_hsa-mir-379	1.27	0.01
hsa-miR-1270_hsa-mir-1270	-1.85	0.01
hsa-let-7e-5p_hsa-let-7e	-1.29	0.01
hsa-miR-874-3p_hsa-mir-874	-1.16	0.02
hsa-miR-651-5p_hsa-mir-651	1.18	0.02
hsa-miR-4326_hsa-mir-4326	1.59	0.02
hsa-miR-548ad-5p_hsa-mir-548ae-2	1.38	0.02
hsa-miR-548ae-5p_hsa-mir-548ae-2	1.38	0.02
hsa-miR-548ad-5p_hsa-mir-548ad	1.34	0.02
hsa-miR-548ae-5p_hsa-mir-548ad	1.34	0.02
hsa-miR-548ad-5p_hsa-mir-548d-1	1.32	0.02
hsa-miR-548ae-5p_hsa-mir-548d-1	1.32	0.02
hsa-miR-548ay-5p_hsa-mir-548d-1	1.32	0.02
hsa-miR-548d-5p_hsa-mir-548d-1	1.32	0.02
hsa-miR-331-5p_hsa-mir-331	1.45	0.02
hsa-miR-21-3p_hsa-mir-21	1.30	0.02
hsa-miR-191-3p_hsa-mir-191	-1.03	0.02
hsa-miR-548ad-5p_hsa-mir-548d-2	1.30	0.02

miRNA_precursor	log (fold change)	*P*-Value
hsa-miR-548ae-5p_hsa-mir-548d-2	1.30	0.02
hsa-miR-548ay-5p_hsa-mir-548d-2	1.30	0.02
hsa-miR-548d-5p_hsa-mir-548d-2	1.30	0.02
hsa-miR-500b-5p_hsa-mir-500a	2.13	0.02
hsa-miR-500b-5p_hsa-mir-500b	2.13	0.02
hsa-miR-615-3p_hsa-mir-615	-1.45	0.02
hsa-miR-1292-5p_hsa-mir-1292	1.06	0.02
hsa-miR-361-3p_hsa-mir-361	-1.19	0.02
hsa-miR-1179_hsa-mir-1179	1.85	0.02
hsa-miR-3158-3p_hsa-mir-3158-1	1.60	0.02
hsa-miR-3158-3p_hsa-mir-3158-2	1.60	0.02
hsa-miR-382-5p_hsa-mir-382	-1.03	0.02
hsa-miR-223-5p_hsa-mir-223	-1.07	0.02
hsa-miR-365a-3p_hsa-mir-365a	1.34	0.02
hsa-miR-365a-3p_hsa-mir-365b	1.34	0.02
hsa-miR-365b-3p_hsa-mir-365a	1.34	0.02
hsa-miR-365b-3p_hsa-mir-365b	1.34	0.02
hsa-miR-6514-5p_hsa-mir-6514	-2.28	0.02
hsa-miR-3928-3p_hsa-mir-3928	-1.27	0.02
hsa-miR-365a-5p_hsa-mir-365a	-2.27	0.02
hsa-miR-548a-3p_hsa-mir-548a-2	1.71	0.02
hsa-miR-548a-3p_hsa-mir-548a-3	1.71	0.02
hsa-miR-550a-3-5p_hsa-mir-550a-3	1.62	0.02
hsa-miR-381-3p_hsa-mir-381	1.21	0.02
hsa-miR-642b-3p_hsa-mir-642b	1.81	0.03
hsa-miR-378a-5p_hsa-mir-378a	1.14	0.03
hsa-miR-6511b-3p_hsa-mir-6511b-1	-1.75	0.03
hsa-miR-154-5p_hsa-mir-154	1.45	0.03
hsa-miR-378i_hsa-mir-378i	1.17	0.03
hsa-miR-4489_hsa-mir-4489	-2.24	0.03
hsa-miR-548a-3p_hsa-mir-548a-1	1.62	0.03
hsa-miR-3614-5p_hsa-mir-3614	-1.76	0.03
hsa-miR-505-3p_hsa-mir-505	1.19	0.03
hsa-miR-452-5p_hsa-mir-452	-1.45	0.03
hsa-miR-330-3p_hsa-mir-330	1.43	0.03
hsa-miR-6511b-3p_hsa-mir-6511b-2	-1.77	0.04
hsa-miR-3150a-3p_hsa-mir-3150a	-2.00	0.04
hsa-miR-185-3p_hsa-mir-185	1.18	0.04
hsa-miR-3143_hsa-mir-3143	1.51	0.04

miRNA_precursor	log (fold change)	*P*-Value
hsa-miR-181c-3p_hsa-mir-181c	1.26	0.04
hsa-miR-2276-3p_hsa-mir-2276	1.48	0.04
hsa-miR-204-3p_hsa-mir-204	1.61	0.04
hsa-miR-181d-5p_hsa-mir-181d	1.24	0.04
hsa-miR-3176_hsa-mir-3176	1.69	0.04
hsa-miR-1343-3p_hsa-mir-1343	-1.76	0.04
hsa-miR-4492_hsa-mir-4492	-1.96	0.04
hsa-miR-509-3p_hsa-mir-509-1	-2.05	0.04
hsa-miR-509-3p_hsa-mir-509-2	-2.05	0.04
hsa-miR-509-3p_hsa-mir-509-3	-2.05	0.04
hsa-miR-6821-5p_hsa-mir-6821	-1.81	0.04
hsa-miR-1250-5p_hsa-mir-1250	1.39	0.046
hsa-miR-365b-5p_hsa-mir-365b	-1.69	0.046
hsa-miR-6764-5p_hsa-mir-6764	-1.69	0.048
hsa-miR-548h-3p_hsa-mir-548h-4	1.33	0.049
hsa-miR-548z_hsa-mir-548h-4	1.33	0.049

Supplementary Table S3. List of Differentially Expressed miRNAs in Premature Thelarche vs. Controls

miRNA_precursor	log (Fold change)	*P*-Value
hsa-miR-22-3p_hsa-mir-22	2.74	8.67E-18
hsa-miR-222-3p_hsa-mir-222	3.14	2.53E-17
hsa-miR-221-3p_hsa-mir-221	2.22	1.13E-14
hsa-miR-20a-5p_hsa-mir-20a	3.15	8.09E-14
hsa-miR-374a-5p_hsa-mir-374a	3.54	1.57E-13
hsa-miR-4429_hsa-mir-4429	3.49	1.48E-12
hsa-miR-1224-5p_hsa-mir-1224	-3.28	2.82E-12
hsa-let-7g-5p_hsa-let-7g	2.40	6.01E-12
hsa-miR-107_hsa-mir-107	1.77	7.85E-12
hsa-miR-15a-5p_hsa-mir-15a	2.01	1.89E-11
hsa-miR-424-5p_hsa-mir-424	2.25	3.40E-11
hsa-miR-139-3p_hsa-mir-139	-2.98	6.90E-10
hsa-miR-363-3p_hsa-mir-363	1.82	6.93E-10
hsa-let-7b-5p_hsa-let-7b	-1.97	1.03E-09
hsa-miR-374b-5p_hsa-mir-374b	3.53	1.94E-09
hsa-miR-22-5p_hsa-mir-22	4.41	8.43E-09
hsa-miR-129-5p_hsa-mir-129-1	-3.43	1.47E-08
hsa-miR-129-5p_hsa-mir-129-2	-3.43	1.47E-08
hsa-miR-15b-5p_hsa-mir-15b	1.74	1.83E-08

miRNA_precursor	log (Fold change)	*P*-Value
hsa-miR-29b-3p_hsa-mir-29b-2	2.91	7.40E-08
hsa-miR-29b-3p_hsa-mir-29b-1	2.91	7.47E-08
hsa-miR-31-5p_hsa-mir-31	4.31	1.14E-07
hsa-miR-27a-3p_hsa-mir-27a	1.44	1.30E-07
hsa-miR-451a_hsa-mir-451a	2.48	1.33E-07
hsa-miR-32-5p_hsa-mir-32	1.88	1.48E-07
hsa-miR-122-5p_hsa-mir-122	-3.33	1.96E-07
hsa-miR-18a-5p_hsa-mir-18a	2.87	2.56E-07
hsa-miR-423-3p_hsa-mir-423	2.02	4.22E-07
hsa-miR-26b-5p_hsa-mir-26b	1.76	5.34E-07
hsa-miR-142-5p_hsa-mir-142	2.56	7.50E-07
hsa-miR-16-2-3p_hsa-mir-16-2	2.06	1.16E-06
hsa-miR-19b-3p_hsa-mir-19b-2	1.90	1.24E-06
hsa-miR-19b-3p_hsa-mir-19b-1	1.90	1.27E-06
hsa-miR-106b-5p_hsa-mir-106b	3.02	2.29E-06
hsa-miR-1294_hsa-mir-1294	-1.81	2.98E-06
hsa-miR-335-5p_hsa-mir-335	1.82	3.13E-06
hsa-let-7c-5p_hsa-let-7c	-1.39	3.60E-06
hsa-miR-181a-5p_hsa-mir-181a-2	2.03	4.25E-06
hsa-miR-181a-5p_hsa-mir-181a-1	2.03	4.28E-06
hsa-miR-30b-5p_hsa-mir-30b	1.78	5.46E-06
hsa-miR-590-3p_hsa-mir-590	2.79	5.85E-06
hsa-miR-499a-5p_hsa-mir-499a	3.20	5.87E-06
hsa-miR-483-5p_hsa-mir-483	-2.10	7.30E-06
hsa-miR-4732-5p_hsa-mir-4732	-1.83	7.77E-06
hsa-miR-4732-3p_hsa-mir-4732	-2.26	8.62E-06
hsa-miR-301a-3p_hsa-mir-301a	2.17	9.06E-06
hsa-miR-885-3p_hsa-mir-885	-5.12	9.23E-06
hsa-miR-760_hsa-mir-760	-1.97	9.51E-06
hsa-miR-17-5p_hsa-mir-17	1.83	1.08E-05
hsa-miR-7-1-3p_hsa-mir-7-1	3.09	1.51E-05
hsa-miR-30a-3p_hsa-mir-30a	-1.81	1.74E-05
hsa-miR-125b-5p_hsa-mir-125b-1	-1.71	1.94E-05
hsa-miR-125b-5p_hsa-mir-125b-2	-1.71	1.95E-05
hsa-miR-5187-5p_hsa-mir-5187	-2.28	2.25E-05
hsa-let-7g-3p_hsa-let-7g	2.63	2.43E-05
hsa-miR-197-5p_hsa-mir-197	-2.79	3.89E-05
hsa-miR-144-3p_hsa-mir-144	2.28	4.05E-05
hsa-miR-2116-3p_hsa-mir-2116	-2.97	4.24E-05

miRNA_precursor	log (Fold change)	*P*-Value
hsa-miR-206_hsa-mir-206	-3.34	4.65E-05
hsa-miR-21-5p_hsa-mir-21	1.84	4.75E-05
hsa-miR-320a-3p_hsa-mir-320a	1.39	5.78E-05
hsa-miR-151a-3p_hsa-mir-151a	-2.08	6.15E-05
hsa-miR-24-3p_hsa-mir-24-2	1.63	6.17E-05
hsa-miR-24-3p_hsa-mir-24-1	1.62	6.43E-05
hsa-miR-1343-3p_hsa-mir-1343	-4.93	6.44E-05
hsa-miR-1277-5p_hsa-mir-1277	2.13	7.52E-05
hsa-miR-432-5p_hsa-mir-432	-2.31	8.17E-05
hsa-miR-19a-3p_hsa-mir-19a	1.90	8.62E-05
hsa-miR-193b-5p_hsa-mir-193b	-2.20	8.75E-05
hsa-miR-125a-5p_hsa-mir-125a	-1.66	8.93E-05
hsa-miR-142-3p_hsa-mir-142	1.57	1.11E-04
hsa-miR-502-3p_hsa-mir-502	1.60	1.18E-04
hsa-miR-103a-3p_hsa-mir-103a-1	1.08	1.30E-04
hsa-miR-103a-3p_hsa-mir-103a-2	1.08	1.30E-04
hsa-miR-7704_hsa-mir-7704	-3.71	1.54E-04
hsa-miR-4669_hsa-mir-4669	-3.65	1.55E-04
hsa-miR-4508_hsa-mir-4508	-1.37	1.63E-04
hsa-miR-3605-3p_hsa-mir-3605	-1.80	1.66E-04
hsa-miR-625-5p_hsa-mir-625	1.54	1.90E-04
hsa-miR-4750-5p_hsa-mir-4750	-3.32	1.94E-04
hsa-miR-1304-3p_hsa-mir-1304	-1.47	2.11E-04
hsa-miR-483-3p_hsa-mir-483	-2.32	2.28E-04
hsa-miR-4446-3p_hsa-mir-4446	-2.97	2.34E-04
hsa-miR-25-3p_hsa-mir-25	-1.31	2.34E-04
hsa-miR-28-5p_hsa-mir-28	2.70	2.52E-04
hsa-miR-30c-2-3p_hsa-mir-30c-2	-4.36	3.12E-04
hsa-miR-1255b-5p_hsa-mir-1255b-1	-2.25	3.58E-04
hsa-miR-1255b-5p_hsa-mir-1255b-2	-2.24	3.62E-04
hsa-miR-30d-5p_hsa-mir-30d	-1.18	4.91E-04
hsa-miR-23a-3p_hsa-mir-23a	1.22	5.42E-04
hsa-miR-486-5p_hsa-mir-486-2	-1.31	5.48E-04
hsa-miR-486-5p_hsa-mir-486-1	-1.31	5.50E-04
hsa-miR-30a-5p_hsa-mir-30a	-1.27	5.78E-04
hsa-miR-99b-5p_hsa-mir-99b	-1.94	6.63E-04
hsa-miR-182-5p_hsa-mir-182	-1.51	7.40E-04
hsa-miR-3154_hsa-mir-3154	-2.87	7.90E-04
hsa-miR-3667-5p_hsa-mir-3667	2.67	9.83E-04

miRNA_precursor	log (Fold change)	*P*-Value
hsa-miR-26a-5p_hsa-mir-26a-2	1.55	1.03E-03
hsa-miR-26a-5p_hsa-mir-26a-1	1.55	1.03E-03
hsa-miR-548ad-5p_hsa-mir-548ae-2	1.92	1.19E-03
hsa-miR-548ae-5p_hsa-mir-548ae-2	1.92	1.19E-03
hsa-miR-6891-5p_hsa-mir-6891	-3.58	1.28E-03
hsa-miR-33a-5p_hsa-mir-33a	2.40	1.32E-03
hsa-miR-30c-5p_hsa-mir-30c-1	1.12	1.33E-03
hsa-miR-30c-5p_hsa-mir-30c-2	1.12	1.35E-03
hsa-miR-339-3p_hsa-mir-339	1.21	1.50E-03
hsa-miR-23b-3p_hsa-mir-23b	1.17	1.59E-03
hsa-miR-1468-5p_hsa-mir-1468	-1.64	1.64E-03
hsa-miR-6807-5p_hsa-mir-6807	-3.13	1.70E-03
hsa-miR-204-3p_hsa-mir-204	2.30	1.91E-03
hsa-miR-92b-3p_hsa-mir-92b	-1.49	1.95E-03
hsa-miR-548ad-5p_hsa-mir-548ad	1.78	1.98E-03
hsa-miR-548ae-5p_hsa-mir-548ad	1.78	1.98E-03
hsa-miR-4516_hsa-mir-4516	-2.71	2.14E-03
hsa-miR-3614-5p_hsa-mir-3614	-3.22	2.23E-03
hsa-miR-10a-3p_hsa-mir-10a	-1.57	2.23E-03
hsa-miR-223-5p_hsa-mir-223	-1.45	2.26E-03
hsa-miR-4467_hsa-mir-4467	-2.55	2.37E-03
hsa-miR-328-3p_hsa-mir-328	-1.07	2.48E-03
hsa-miR-548ad-5p_hsa-mir-548ay	1.77	2.56E-03
hsa-miR-548ae-5p_hsa-mir-548ay	1.77	2.56E-03
hsa-miR-548ay-5p_hsa-mir-548ay	1.77	2.56E-03
hsa-miR-6780a-5p_hsa-mir-6780a	-1.89	2.67E-03
hsa-miR-375-3p_hsa-mir-375	-1.60	2.78E-03
hsa-miR-183-5p_hsa-mir-183	-1.22	2.83E-03
hsa-miR-6514-5p_hsa-mir-6514	-3.58	2.91E-03
hsa-miR-320e_hsa-mir-320e	1.50	2.93E-03
hsa-miR-6511a-3p_hsa-mir-6511a-1	-1.85	3.44E-03
hsa-miR-6511a-3p_hsa-mir-6511a-2	-1.85	3.44E-03
hsa-miR-6511a-3p_hsa-mir-6511a-3	-1.85	3.44E-03
hsa-miR-6511a-3p_hsa-mir-6511a-4	-1.85	3.44E-03
hsa-miR-130a-3p_hsa-mir-130a	1.00	3.54E-03
hsa-miR-376a-3p_hsa-mir-376a-1	1.71	3.76E-03
hsa-miR-376a-3p_hsa-mir-376a-2	1.71	3.76E-03
hsa-miR-370-3p_hsa-mir-370	-1.55	3.82E-03
hsa-miR-6734-5p_hsa-mir-6734	-1.34	4.22E-03

miRNA_precursor	log (Fold change)	*P*-Value
hsa-miR-548ad-5p_hsa-mir-548d-1	1.66	4.32E-03
hsa-miR-548ad-5p_hsa-mir-548d-2	1.66	4.32E-03
hsa-miR-548ae-5p_hsa-mir-548d-1	1.66	4.32E-03
hsa-miR-548ae-5p_hsa-mir-548d-2	1.66	4.32E-03
hsa-miR-548ay-5p_hsa-mir-548d-1	1.66	4.32E-03
hsa-miR-548ay-5p_hsa-mir-548d-2	1.66	4.32E-03
hsa-miR-548d-5p_hsa-mir-548d-1	1.66	4.32E-03
hsa-miR-548d-5p_hsa-mir-548d-2	1.66	4.32E-03
hsa-miR-143-5p_hsa-mir-143	2.30	4.50E-03
hsa-miR-3198_hsa-mir-3198-1	-2.73	4.71E-03
hsa-miR-3198_hsa-mir-3198-2	-2.73	4.71E-03
hsa-miR-6793-5p_hsa-mir-6793	-2.99	4.71E-03
hsa-miR-874-5p_hsa-mir-874	2.32	4.77E-03
hsa-miR-664a-5p_hsa-mir-664a	-1.24	4.83E-03
hsa-miR-548h-3p_hsa-mir-548h-4	2.14	4.87E-03
hsa-miR-548z_hsa-mir-548h-4	2.14	4.87E-03
hsa-miR-664b-5p_hsa-mir-664b	-1.62	5.11E-03
hsa-miR-196a-5p_hsa-mir-196a-2	-1.72	5.18E-03
hsa-miR-127-3p_hsa-mir-127	-1.71	5.41E-03
hsa-miR-548h-3p_hsa-mir-548z	2.04	6.47E-03
hsa-miR-548z_hsa-mir-548z	2.04	6.47E-03
hsa-miR-6721-5p_hsa-mir-6721	-2.96	6.68E-03
hsa-miR-1307-5p_hsa-mir-1307	1.14	6.68E-03
hsa-miR-140-5p_hsa-mir-140	1.22	6.87E-03
hsa-miR-195-3p_hsa-mir-195	-2.68	7.09E-03
hsa-miR-145-3p_hsa-mir-145	2.04	7.21E-03
hsa-miR-337-5p_hsa-mir-337	1.73	7.26E-03
hsa-miR-1908-5p_hsa-mir-1908	-1.79	7.44E-03
hsa-miR-98-5p_hsa-mir-98	1.00	7.54E-03
hsa-miR-3150b-3p_hsa-mir-3150b	-2.12	7.61E-03
hsa-miR-4665-5p_hsa-mir-4665	-3.11	7.64E-03
hsa-miR-196a-5p_hsa-mir-196a-1	-1.56	7.66E-03
hsa-miR-574-3p_hsa-mir-574	-1.11	8.15E-03
hsa-miR-365a-5p_hsa-mir-365a	-2.98	8.89E-03
hsa-miR-642a-5p_hsa-mir-642a	1.81	9.03E-03
hsa-miR-4286_hsa-mir-4286	1.68	9.04E-03
hsa-miR-652-3p_hsa-mir-652	1.27	9.35E-03
hsa-miR-379-5p_hsa-mir-379	1.38	9.56E-03
hsa-miR-3960_hsa-mir-3960	-1.88	9.60E-03

miRNA_precursor	log (Fold change)	*P*-Value
hsa-miR-4488_hsa-mir-4488	2.35	9.80E-03
hsa-miR-29a-3p_hsa-mir-29a	1.41	9.81E-03
hsa-miR-146a-5p_hsa-mir-146a	1.14	0.01
hsa-miR-873-5p_hsa-mir-873	2.06	0.01
hsa-miR-141-3p_hsa-mir-141	1.22	0.01
hsa-miR-1270_hsa-mir-1270	-1.89	0.01
hsa-miR-6779-5p_hsa-mir-6779	-2.89	0.01
hsa-miR-125b-1-3p_hsa-mir-125b-1	-2.49	0.01
hsa-miR-382-5p_hsa-mir-382	-1.30	0.01
hsa-miR-642b-3p_hsa-mir-642b	2.42	0.01
hsa-miR-433-3p_hsa-mir-433	-2.97	0.01
hsa-let-7e-5p_hsa-let-7e	-1.33	0.01
hsa-miR-3127-5p_hsa-mir-3127	-1.95	0.01
hsa-miR-484_hsa-mir-484	1.26	0.01
hsa-miR-548am-5p_hsa-mir-548am	1.75	0.01
hsa-miR-548am-5p_hsa-mir-548c	1.75	0.01
hsa-miR-548am-5p_hsa-mir-548o-2	1.75	0.01
hsa-miR-548au-5p_hsa-mir-548am	1.75	0.01
hsa-miR-548au-5p_hsa-mir-548c	1.75	0.01
hsa-miR-548au-5p_hsa-mir-548o-2	1.75	0.01
hsa-miR-548c-5p_hsa-mir-548am	1.75	0.01
hsa-miR-548c-5p_hsa-mir-548c	1.75	0.01
hsa-miR-548c-5p_hsa-mir-548o-2	1.75	0.01
hsa-miR-548o-5p_hsa-mir-548am	1.75	0.01
hsa-miR-548o-5p_hsa-mir-548c	1.75	0.01
hsa-miR-548o-5p_hsa-mir-548o-2	1.75	0.01
hsa-miR-1268a_hsa-mir-1268a	-2.35	0.01
hsa-miR-3682-3p_hsa-mir-3682	-2.64	0.02
hsa-miR-3913-5p_hsa-mir-3913-1	-1.40	0.02
hsa-miR-3913-5p_hsa-mir-3913-2	-1.40	0.02
hsa-miR-450b-5p_hsa-mir-450b	1.62	0.02
hsa-miR-668-3p_hsa-mir-668	-2.40	0.02
hsa-let-7i-3p_hsa-let-7i	1.42	0.02
hsa-miR-937-3p_hsa-mir-937	-2.93	0.02
hsa-miR-3187-3p_hsa-mir-3187	-1.14	0.02
hsa-miR-627-5p_hsa-mir-627	1.73	0.02
hsa-miR-7706_hsa-mir-7706	-1.74	0.02
hsa-miR-1273h-5p_hsa-mir-1273h	-2.52	0.02
hsa-miR-146b-5p_hsa-mir-146b	1.21	0.02

miRNA_precursor	log (Fold change)	*P*-Value
hsa-miR-132-5p_hsa-mir-132	2.04	0.02
hsa-miR-377-3p_hsa-mir-377	1.91	0.02
hsa-miR-211-5p_hsa-mir-211	-2.54	0.02
hsa-miR-320d_hsa-mir-320d-2	1.42	0.02
hsa-miR-6731-5p_hsa-mir-6731	-1.87	0.02
hsa-miR-92b-5p_hsa-mir-92b	-1.33	0.02
hsa-miR-5196-3p_hsa-mir-5196	-2.37	0.02
hsa-miR-224-5p_hsa-mir-224	-1.67	0.02
hsa-miR-191-3p_hsa-mir-191	-1.16	0.02
hsa-miR-181d-5p_hsa-mir-181d	1.46	0.02
hsa-miR-505-5p_hsa-mir-505	-1.22	0.02
hsa-miR-485-5p_hsa-mir-485	-1.37	0.02
hsa-miR-6805-5p_hsa-mir-6805	-1.41	0.02
hsa-miR-1275_hsa-mir-1275	-2.45	0.03
hsa-miR-452-5p_hsa-mir-452	-1.67	0.03
hsa-miR-1290_hsa-mir-1290	-1.36	0.03
hsa-miR-9-3p_hsa-mir-9-1	1.38	0.03
hsa-miR-9-3p_hsa-mir-9-2	1.38	0.03
hsa-miR-9-3p_hsa-mir-9-3	1.38	0.03
hsa-miR-1179_hsa-mir-1179	1.87	0.03
hsa-miR-184_hsa-mir-184	-3.26	0.03
hsa-miR-320d_hsa-mir-320d-1	1.38	0.03
hsa-miR-4433a-3p_hsa-mir-4433a	1.82	0.03
hsa-miR-2355-3p_hsa-mir-2355	1.53	0.03
hsa-miR-6767-5p_hsa-mir-6767	-1.68	0.03
hsa-miR-424-3p_hsa-mir-424	1.72	0.03
hsa-miR-6741-5p_hsa-mir-6741	-1.94	0.03
hsa-miR-1250-5p_hsa-mir-1250	1.73	0.03
hsa-miR-494-3p_hsa-mir-494	1.27	0.03
hsa-miR-376c-3p_hsa-mir-376c	1.68	0.03
hsa-miR-101-5p_hsa-mir-101-1	1.51	0.03
hsa-miR-10399-5p_hsa-mir-10399	1.65	0.03
hsa-miR-548a-3p_hsa-mir-548a-1	1.73	0.03
hsa-miR-548a-3p_hsa-mir-548a-2	1.73	0.03
hsa-miR-548a-3p_hsa-mir-548a-3	1.73	0.03
hsa-miR-200b-3p_hsa-mir-200b	1.40	0.03
hsa-miR-320b_hsa-mir-320b-2	1.10	0.03
hsa-miR-320b_hsa-mir-320b-1	1.10	0.03
hsa-miR-1299_hsa-mir-1299	-2.01	0.04

miRNA_precursor	log (Fold change)	*P*-Value
hsa-miR-4685-3p_hsa-mir-4685	1.67	0.04
hsa-miR-4433a-5p_hsa-mir-4433a	-1.80	0.04
hsa-miR-4511_hsa-mir-4511	-1.82	0.04
hsa-miR-664b-3p_hsa-mir-664b	-2.05	0.04
hsa-miR-214-3p_hsa-mir-214	1.45	0.04
hsa-miR-7977_hsa-mir-7977	1.68	0.04
hsa-miR-7976_hsa-mir-7976	-1.06	0.04
hsa-miR-29c-5p_hsa-mir-29c	1.20	0.04
hsa-miR-651-5p_hsa-mir-651	1.24	0.04
hsa-miR-4454_hsa-mir-4454	1.64	0.04
hsa-miR-202-3p_hsa-mir-202	-1.57	0.04
hsa-miR-615-3p_hsa-mir-615	-1.31	0.04
hsa-miR-548ap-3p_hsa-mir-548aa-1	1.94	0.04
hsa-miR-548ap-3p_hsa-mir-548aa-2	1.94	0.04
hsa-miR-548ap-3p_hsa-mir-548t	1.94	0.04
hsa-miR-6852-5p_hsa-mir-6852	-2.04	0.04
hsa-miR-95-3p_hsa-mir-95	1.06	0.04
hsa-miR-18a-3p_hsa-mir-18a	1.35	0.045
hsa-miR-636_hsa-mir-636	-1.16	0.045
hsa-miR-3124-5p_hsa-mir-3124	-1.79	0.046
hsa-miR-409-3p_hsa-mir-409	-1.09	0.047
hsa-miR-320c_hsa-mir-320c-2	1.09	0.047
hsa-miR-582-3p_hsa-mir-582	-1.68	0.047
hsa-miR-6877-5p_hsa-mir-6877	1.52	0.047
hsa-miR-320c_hsa-mir-320c-1	1.08	0.048
hsa-miR-10527-5p_hsa-mir-10527	1.05	0.049
